# Interactions between shape-persistent macromolecules as probed by AFM

**DOI:** 10.3762/bjoc.13.95

**Published:** 2017-05-18

**Authors:** Johanna Blass, Jessica Brunke, Franziska Emmerich, Cédric Przybylski, Vasil M Garamus, Artem Feoktystov, Roland Bennewitz, Gerhard Wenz, Marcel Albrecht

**Affiliations:** 1 INM-Leibniz-Institute for New Materials, Saarland University, Campus D 2.2, D-66123 Saarbrücken, Germany; 2 Physics Department, Saarland University, Campus D 2.2, D-66123 Saarbrücken, Germany; 3 Organic Macromolecular Chemistry, Saarland University, Campus C 4.2, D-66123 Saarbrücken, Germany; 4 UPMC, IPCM-CNRS UMR 8232, Sorbonne Universités, 75252 Paris Cedex 05, France; 5 Helmholtz-Zentrum Geesthacht (HZG), Centre for Materials and Costal Research, Max-Planck-Str. 1, 21502 Geesthacht, Germany; 6 Jülich Centre for Neutron Science (JCNS) at Heinz Maier-Leibnitz Zentrum (MLZ), Forschungszentrum Jülich GmbH, Lichtenbergstr. 1, 85748 Garching, Germany

**Keywords:** AFM, cyclodextrin, inclusion complexes, molecular recognition, polyconjugated polymers, shape persistent polymers

## Abstract

Water-soluble shape-persistent cyclodextrin (CD) polymers with amino-functionalized end groups were prepared starting from diacetylene-modified cyclodextrin monomers by a combined Glaser coupling/click chemistry approach. Structural perfection of the neutral CD polymers and inclusion complex formation with ditopic and monotopic guest molecules were proven by MALDI–TOF and UV–vis measurements. Small-angle neutron and X-ray (SANS/SAXS) scattering experiments confirm the stiffness of the polymer chains with an apparent contour length of about 130 Å. Surface modification of planar silicon wafers as well as AFM tips was realized by covalent bound formation between the terminal amino groups of the CD polymer and a reactive isothiocyanate–silane monolayer. Atomic force measurements of CD polymer decorated surfaces show enhanced supramolecular interaction energies which can be attributed to multiple inclusion complexes based on the rigidity of the polymer backbone and the regular configuration of the CD moieties. Depending on the geometrical configuration of attachment anisotropic adhesion characteristics of the polymer system can be distinguished between a peeling and a shearing mechanism.

## Introduction

Shape-persistence is an important key feature in self-organisation strategies of supramolecular building blocks resulting in high structural perfection of the obtained molecular assemblies [1], such as shape persistent macrocycles, cage compounds or rotaxanes [2–4]. Especially shape-persistent polymers are of significant scientific interest as their defined structural characteristics offer various applications as sensor materials, biomimetic filaments or organic electronics [5–7]. Furthermore, compared to polymers with flexible chains, shape persistent macromolecules with high structural rigidity are able to form stable aggregates based on multiple supramolecular interactions, which can be detected and quantified without the presence of side effects, such as self-passivation or coiling processes. Dendrimers, nanoparticles and shape-persistent polymers had been previously discussed as scaffolds for the design of multiple ligands of high affinity [8]. Nevertheless, well-defined model systems in which the influence of rigidity and regularity on cooperativity of binding was systematically investigated have not been reported so far.

Rigid linear polymers have been considered as suitable scaffolds for the design of supramolecular systems showing multiple interactions. A high rigidity of the macromolecule is maintained by rigid, linear repeat units, such as *trans*-ethenylene, ethynylene, or *p*-phenylene moieties. The observed persistence lengths of polyconjugated polymers ranged from 6 to 16 nm, depending on the side groups and the method of determination [9–11].

**Figure 1:**
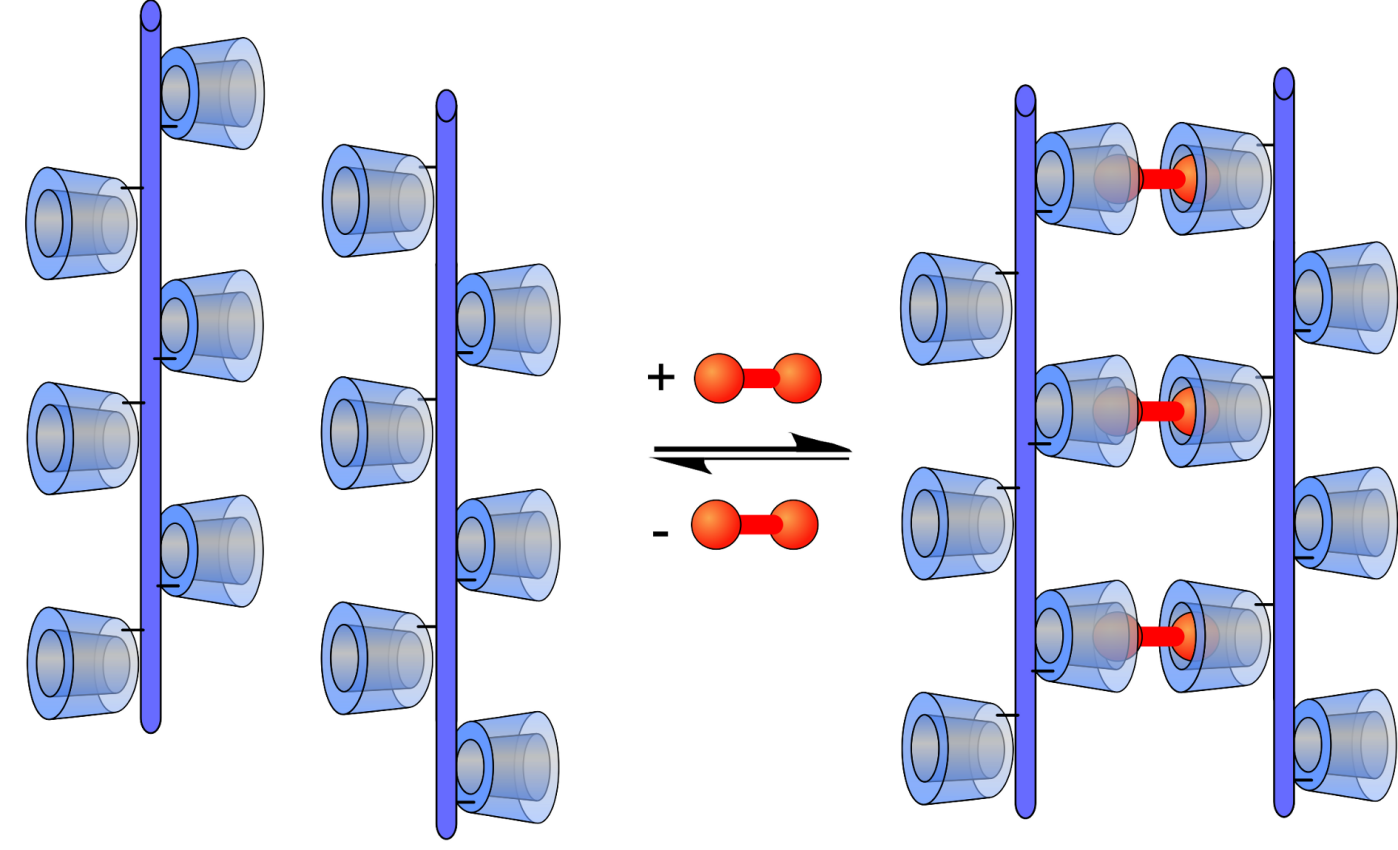
Interaction of a shape-persistent CD polymer with ditopic guests.

Among many supramolecular interactions, such as hydrogen bonding, π–π-interactions or hydrophobic host–guest interactions [12–16], the interactions of cyclodextrins (CDs) with hydrophobic guest molecules are of special interest, since CDs are readily available bio-based materials and interactions take place under physiological conditions [17]. CDs are ideal candidates for the investigation of multivalent interactions as they combine high affinities with a versatile integrability in macromolecular systems [18]. CDs have already been employed for the construction of supramolecular polymers [19–21], supramolecular hydrogels [22–23], molecular printboards [24–25] or multivalent interfaces [26–28] with tunable chemical and physical properties. Herein, for the first time, we present studies concerning the synthesis of shape-persistent CD polymers to investigate multivalent binding with ditopic guest molecules on the molecular level (Figure 1). The ditopic guest (shown in red colour) should act as a connector between opposing CD moieties.

Only a few examples of shape-persistent CD polymers have been reported so far, including CD-modified conjugated oligomers and polymers composed of rigid phenylene ethynylene (PPE) structure units which are able to form self-inclusion complexes with tunable electrochemical properties [29–35]. The synthesis of PPE, in which two β-CD rings were attached to every second phenylene group, was described by Ogoshi et al. [36] using a Sonogashira–Hagiwara coupling. We preferred a poly-phenylene-butadiynylene backbone, synthesized by a Glaser–Eglington coupling, since the repeating unit is long enough (*l* = 0.944 nm) to allow the connection of one CD moiety at each phenylene unit. Based on the stiffness of the polymer chain self-passivation of CD polymer modified surfaces is reduced to a minimum. Furthermore, the ethynyl end groups are easily functionalized by click chemistry.

Isothermal titration calorimetry (ITC), fluorescence spectroscopy, quartz crystal microbalance (QCM), surface plasmon resonance (SPR) and atomic force microscopy (AFM) have been employed to quantify the strength of the multivalent interactions [8]. Because binding affinities can be very high for multivalent supramolecular systems, the constituents are commonly used in low equilibrium concentrations. Since AFM even allows the investigation of single molecules, such as DNA [37–38] or molecular self-assembling based on “Dip-Pen” nanolithography [39], it was chosen as the most reliable technique to probe highly cooperative recognition processes.

**Figure 2:**
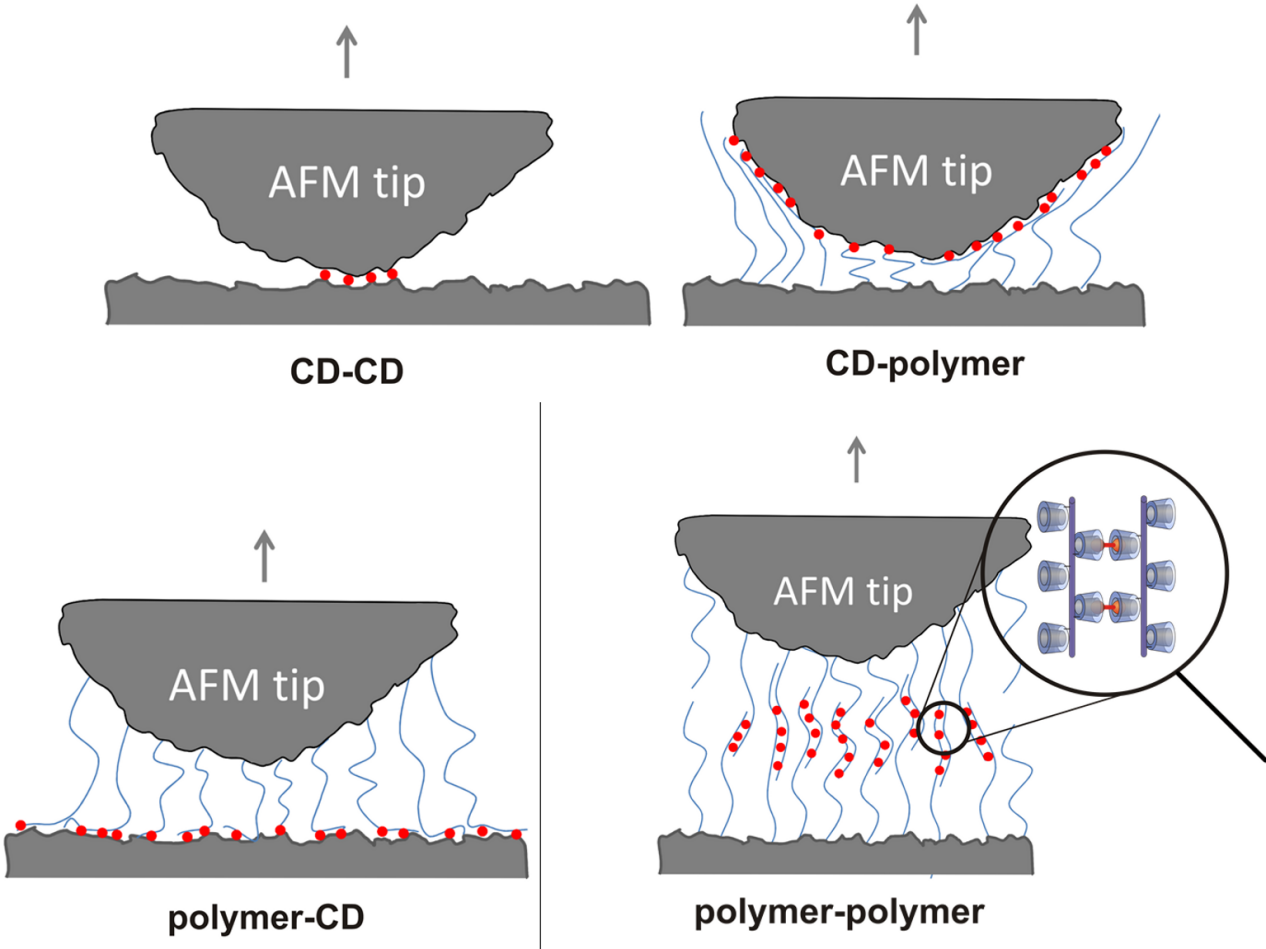
Schematic representation of tip and surface modifications realized in this study (bottom). Blue lines symbolize the CD polymers, red circles the complexed ditopic linkers.

The investigation of cooperativity of multiple host–guest interactions using AFM has been reported by several groups [40–45]. Huskens and co-workers measured the supramolecular interactions between a β-CD-modified planar surface and mono-, di- and trivalent adamantane guest molecules attached to an AFM tip and found enhancement factors up to 2, depending on the force loading rate [46]. We have previously explored the adhesion characteristics of dense CD layers on an AFM tip and a planar silicon surface connected by various ditopic linker molecules. In this system we were able to switch adhesion and friction by applying external stimuli onto the responsive ditopic linkers [47–49]. In contrast to previous work our molecular toolkit, based on ditopic connector molecules, allows the independent determination of unspecific interactions between CD polymers at tip and planar surface as well as the specific interactions to ditopic connector molecules. In the following, we describe the first example of multivalent interaction of ditopic guest molecules with shape-persistent CD polymers covalently attached to an AFM tip and a planar surface. Nano force measurements between CD and CD polymer, CD polymer and CD, and CD and CD at the tip and the planar surface, respectively, exerted by the adamantane ditopic connector molecules were systematically investigated. All four configurations are schematically depicted in Figure 2.

## Results and Discussion

### Synthesis of the shape-persistent CD polymer

**Scheme 1:**
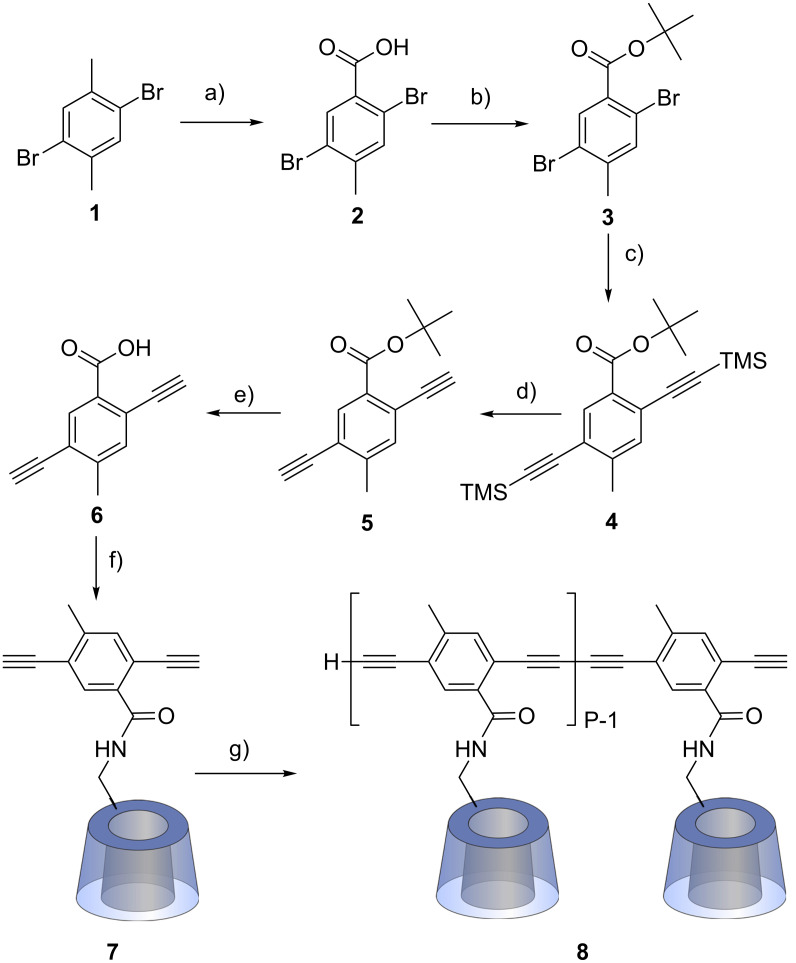
Synthesis of the CD polymer. a) conc. HNO_3_, reflux, 6 d; b) *tert*-butanol, cat. H_2_SO_4_, MgSO_4_, CH_2_Cl_2_, rt, sealed vessel, 4 d; c) TMSA, PdCl_2_ (10 mol %), CuI (5 mol %), PPh_3_ (0.5 equiv), Et_3_N, 80 °C, 48 h; d) TBAF, THF, −20 °C, 30 min; e) TFA, CH_2_Cl_2_, rt, 18 h; f) 6-monoamino-6-deoxy-ß-CD, DCC, HOBt, DMF, rt, 8 d; g) cat. CuCl, cat. Cu(OAc)_2_, pyridine, 60 °C, 24 h.

Our synthetic approach for the preparation of modified poly(phenylene butadiynylene)s bearing one CD molecule per repeat unit started from 2,5-dibromo-4-methylbenzoic acid (**2**) [50–51], which was esterified to **3** with *tert*-butanol catalyzed by H_2_SO_4_ (Scheme 1). The TMS-protected diacetylene derivative **4** was prepared by Sonogashira reaction of **3** with trimethylsilylacetylene. Subsequent deprotection of the TMS groups using tetra-*n*-butylammonium fluoride and saponification of the *tert*-butyl ester with trifluoroacetic acid resulted in the corresponding benzoic acid **6**. The latter was coupled to 6-monoamino-6-deoxy-β-CD [52] using *N,N’*-dicyclohexyl-carbodiimide (DCC) and 1-hydroxybenzotriazole (HOBt) applying a procedure known for terephthalic acid [53]. The resulting product, monomer **7**, was easily isolated due to its low solubility in water which was attributed to self-inclusion between hydrophobic phenyl moieties and β-CD rings leading to daisy chains [54].

**Figure 3:**
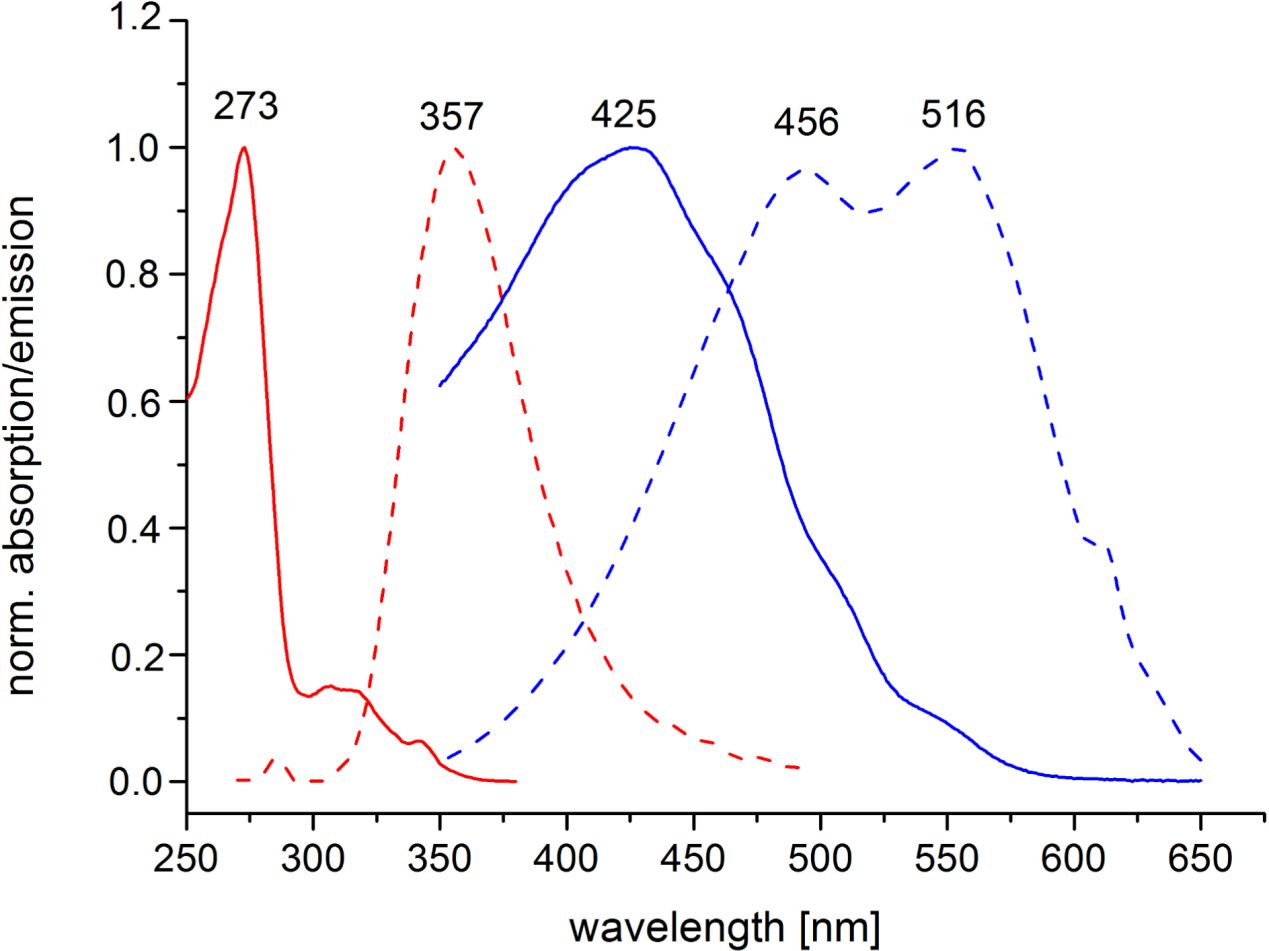
Absorption spectra of monomer **7** (solid red line) and polymer **8** (solid blue line) in water. Emission spectra of monomer **7** (dotted red line) and polymer **8** (dotted blue line) in water excited at 290 nm and 335 nm, respectively.

The polymerization of **7** was performed through Glaser coupling in pyridine catalyzed by Cu(I)/Cu(II). After removal of low molecular weight material by ultrafiltration polymer **8** was isolated as a light orange solid in 91% yield. Polyrotaxane formation, which might prevent the accessibility of the CD-moieties located on the polymer backbone, was avoided by the presence of pyridine as a non-polar solvent. Both NOESY NMR experiments and circular dichroism (results not shown) do not indicate any significant interaction of the CDs and the aromatic backbone. Compared to monomer **7**, peak broadening and the disappearance of the ^1^H NMR signals of the acetylene protons at 4.54 and 4.36 ppm indicate the formation of polymer **8**. The presence of the conjugated backbone was confirmed by UV–vis and fluorescence measurements in water. Compared to **7**, a characteristic bathochromic shift could be observed both in the absorption and emission spectra of polymer **8** (Figure 3) showing the presence of the extended polyconjugated π-system.

**Figure 4:**
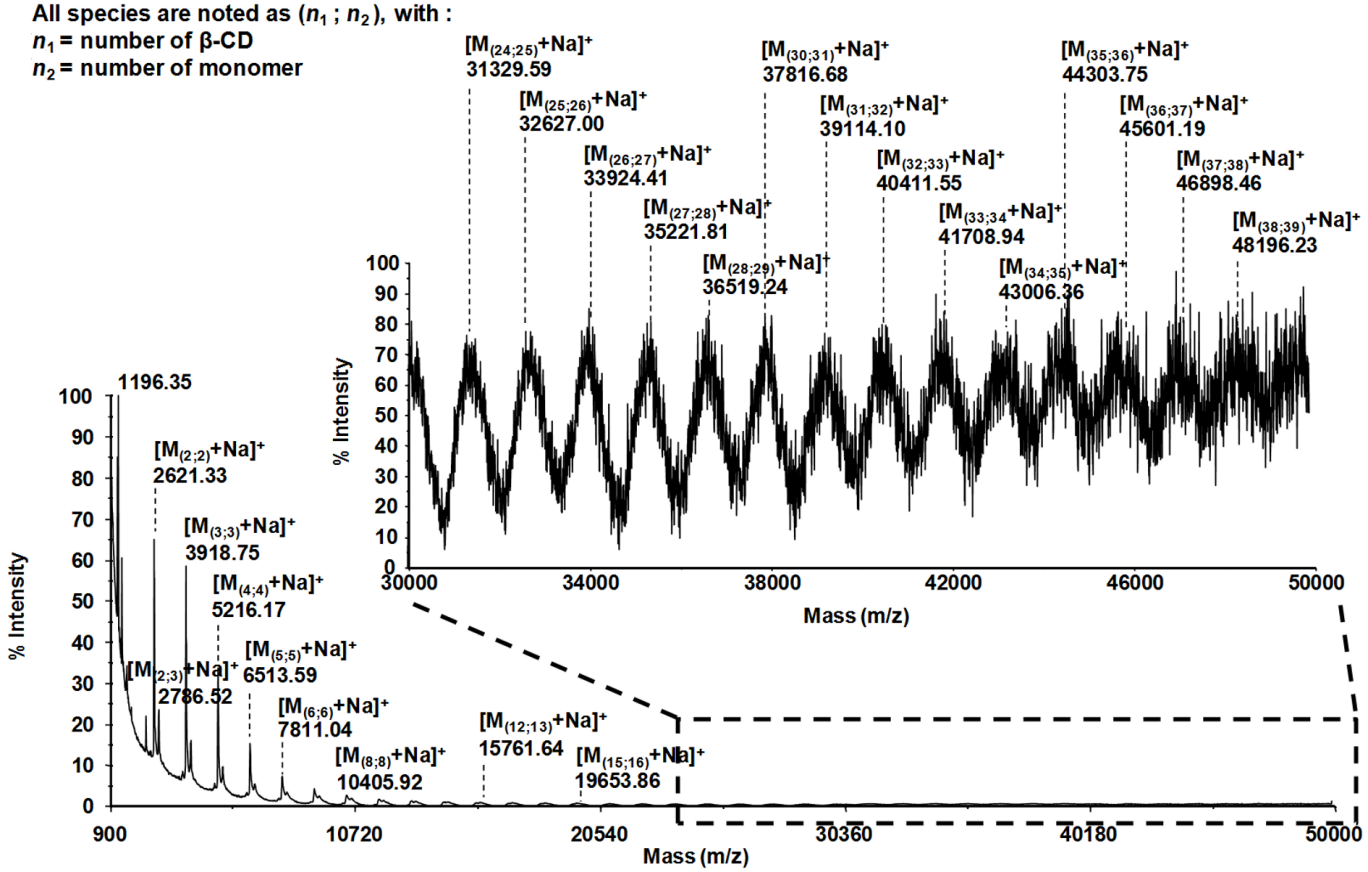
Positive linear MALDI–TOF spectrum of polymer **8** using HABA/TMG_2_ matrix.

Quantitative information about the molecular weight distribution of **8** was obtained by MALDI–TOF measurements using an ionic liquid matrix (HABA/TMG_2_) [55]. A representative MALDI spectrum, shown in Figure 4, exhibits a wide range of broad signals starting from the signal of the dimer at *m*/*z* 2,621.33 Da detected as [M + Na]^+^ and ending at the 38mer at *m*/*z* 48,196.23 Da for a S/N ratio ≥3, with an average 1297.4 mass units shift corresponding to one additional repeating unit. Among each discrete envelope, one to three supplementary ions, have been detected with a constant 165.2 mass unit shift, revealing the presence of small quantities of the repeat unit originating from unmodified benzoic acid derivative **6**, e.g., at 2,621.33 and 2,786.52 Da (Figure 4). The MS analysis reveals the high structural perfection of the polymer **8** where at most one CD entity per polymer molecule is missing. Integration of the relative distribution of the most intense ions of each population allowed to estimate both the number average molecular weight, *M*_n_, and the mass average molecular weight, *M*_w_, of 8,765.77 Da, and 22,023.56 Da, respectively. These values result in a polydispersity index PDI = *M*_w_/*M*_n_ of 2.59 typical for normal distributions. From the value of *M*_w_ an average contour length *L* = 17 nm of the macromolecule was calculated. A more detailed analysis of the MS data is provided in Supporting Information File 1.

### SANS and SAXS measurements of the CD polymer

**Figure 5:**
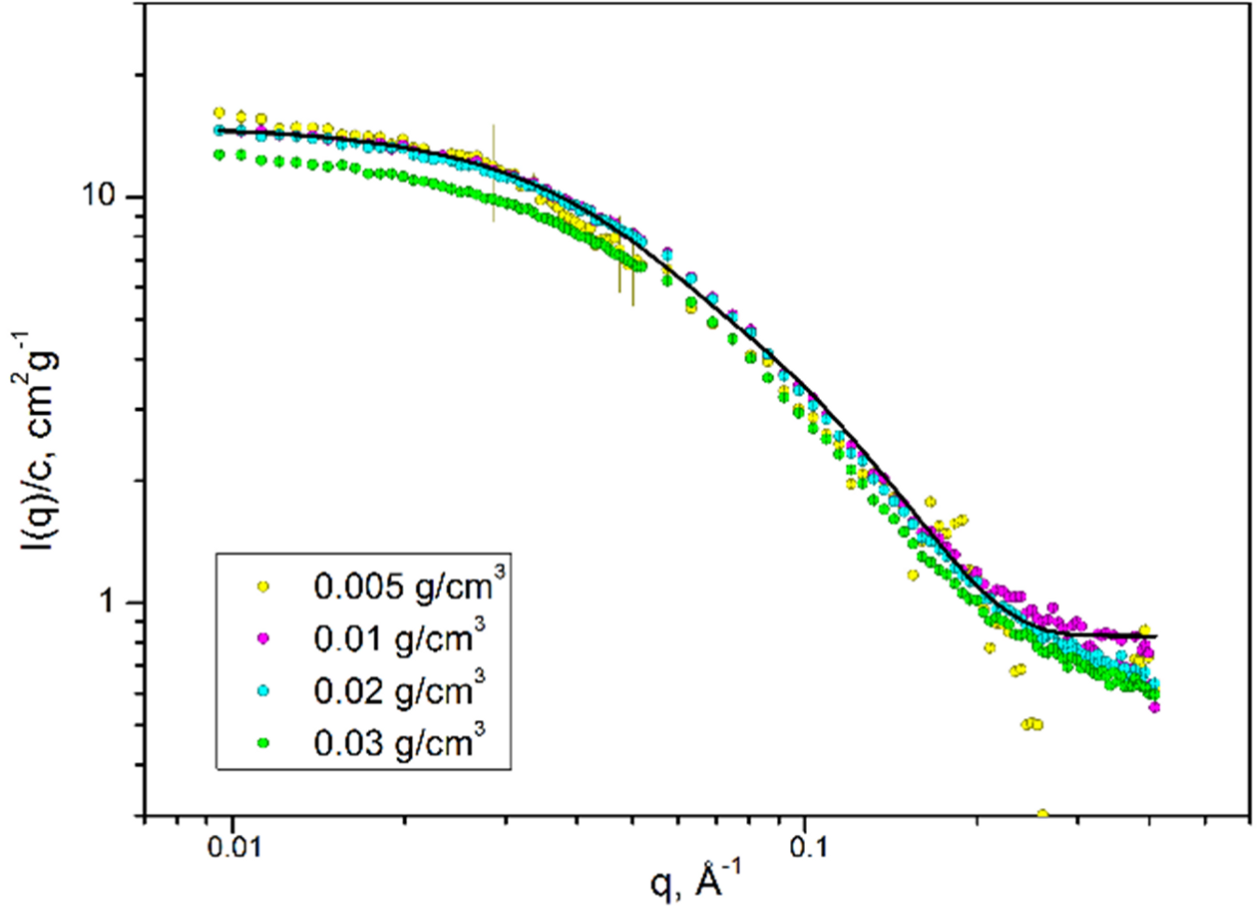
SANS data for polymer **8** and fit by cylindrical model (solid line).

Structural characteristics of the CD polymer **8** have been investigated by small-angle neutron and X-ray scattering experiments (SANS/SAXS). SANS data (KWS-1, JCNS at Heinz Maier-Leibnitz Zentrum [56]) for a polymer concentration range from 0.005 to 0.03 g/cm^3^ are presented in Figure 5. SANS intensities are normalized to polymer concentration and therefore scattering intensities depend on polymer chain mass (or mass of chain aggregates), square of scattering contrast, conformation of polymer chain, and interaction between the chains (aggregates). There are only minor differences in scattering for concentrations up to 0.02 g/cm^3^ indicating no significant aggregation between polymer chains with increasing concentration which would lead to highly ordered polymer species. The decrease of scattering intensity for the highest concentration of 0.03 g/cm^3^ can be attributed to interaction of polymer chains. The SAXS curve measured at 0.03 g/mL shows a similar shape as the neutron data (Supporting Information File 1, Figure S1).

The low-*q* range of scattering data has been analyzed with a Debye function. The apparent radius of gyration *R*_g,app_ and the scattering at “zero angle”, *I*(0), were obtained by fitting the scattering data for *q* < 0.02 Å^−1^ [57]:

[1]
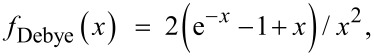


where *x*= *q^2 ^R*_g,app_^2^. The scattering intensity is given by

[2]
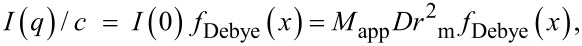


where the apparent molar weight, *M*_app_, is connected with the real molar weight, *M*, via a structure factor *S*(0) (interaction among polymer chains) as *M* × *S*(0) = *M*_app_ and Δ*ρ*_m_ is the difference in neutron scattering length density between polymer and solvent normalized to the density of polymer. The local structure of the polymer cylindrical cross-section was extracted by applying indirect Fourier transformation (IFT) [58] to the experimental data from the high-*q* range. Detailed information applying this method is presented in Supporting Information File 1. The resulting parameters for the concentration dependence of *I*(0), scattering at “zero angle” of a cylindrical cross-section of polymer *I*_CS_(0), radius of gyration *R*_g,app_, and radius of gyration of a cylindrical cross-section *R*_g_*_,_*_CS_ are presented in Table 1. The ratio between *I*(0) and *I*_CS_(0) provides the apparent contour length of the polymer chain. SAXS and SANS indicate a contour length of 130–160 Å, i.e., 15 monomer units with the length of one unit of *L*_mon_ = 9.2 Å and the chemical composition C_54_H_75_NO_35_ (molecular weight 1298.17 g/mol). *R*_g_*_,_*_CS_ has been used to calculate the cross-section diameter of the homogeneous cylinder to be 30 Å.

**Table 1:** Structural parameters of polymer **8** (apparent radius of gyration, scattering at zero angle, radius of gyration of polymer cross-section, scattering at zero angle of polymer cross-section, apparent contour length obtained from the ratio between *I*(0) and *I*_CS_(0), and calculated apparent mass of polymer **8**, obtained from the length of monomer unit *M*_app_ = *M*_mon_ × *L*_app_/*L*_mon_).

Conc, g/mL	*R*_g,app_, Å	*I*(0), cm^2^·g^−1^	*R*_g,CS_, Å	*I*_CS_(0), Å^−1^·cm^2^·g^−1^	*L*_app_, Å	*M*_app_, kDa

0.03 (SAXS)	38.0 ± 1.5	680 ± 10 a.u.	10.2 ± 0.5	5.23 ± 0.05 a.u.	130	18
0.005	37.4 ± 3.5	16.2 ± 0.3	9.8 ± 0.5	0.099 ± 0.002	164	23
0.01	31.6 ± 2.5	15.0 ± 0.2	9.7 ± 0.5	0.119 ± 0.002	126	18
0.02	32.7 ± 1.6	14.9 ± 0.2	9.5 ± 0.5	0.117 ± 0.002	127	18
0.03	34.6 ± 1.5	13.0 ± 0.1	9.4 ± 0.5	0.100 ± 0.002	130	18

Scattering intensities do not change significantly with concentration indicating that the value of *S*(0) is close to 1. We consider the values for *L*_app_ and *M*_app_ as lower limits. They are probably affected by the inexact determination of the scattering contrast.

The apparent mass and contour length of polymer **8** with values of about 18 kDa and 130–160 Å are in the same range as those obtained by MALDI measurements (*M*_w_ = 22 kDa, *L* = 170 Å) and confirm the structural characteristics of the stiff CD polymer.

The flexibility of chains of polymer **8** was determined by means of a Holtzer plot [59]. Detailed information and the corresponding data are presented in Supporting Information File 1. The absence of a characteristic inflection point, where the scattering intensity changes from *q*^−1^ as for rigid cylinder to *q*^−2^ (or to *q*^−5/3^ when self-avoidance is important) as for flexible chains, indicates that polymer chains are short and rigid, i.e., that the persistence length is of the same order as the contour length of the polymer.

The SAXS data has been analyzed by models representing the expected shape of polymers. It was assumed that there is no interaction between aggregates, which means that the scattering intensities depend only on the size and shape of the aggregates [60]. Details are shown in Supporting Information File 1.

The scattering data could be described (Figure 5 above and Figure S1 in Supporting Information File 1) by a population of rigid cylinders of length 110 ± 5 Å and radius of cross-section of 12 ± 2 Å. Neglecting the interaction between polymer chains in the model leads to the slightly lower length values.

### Complexation of monotopic and ditopic guests

**Scheme 2:**
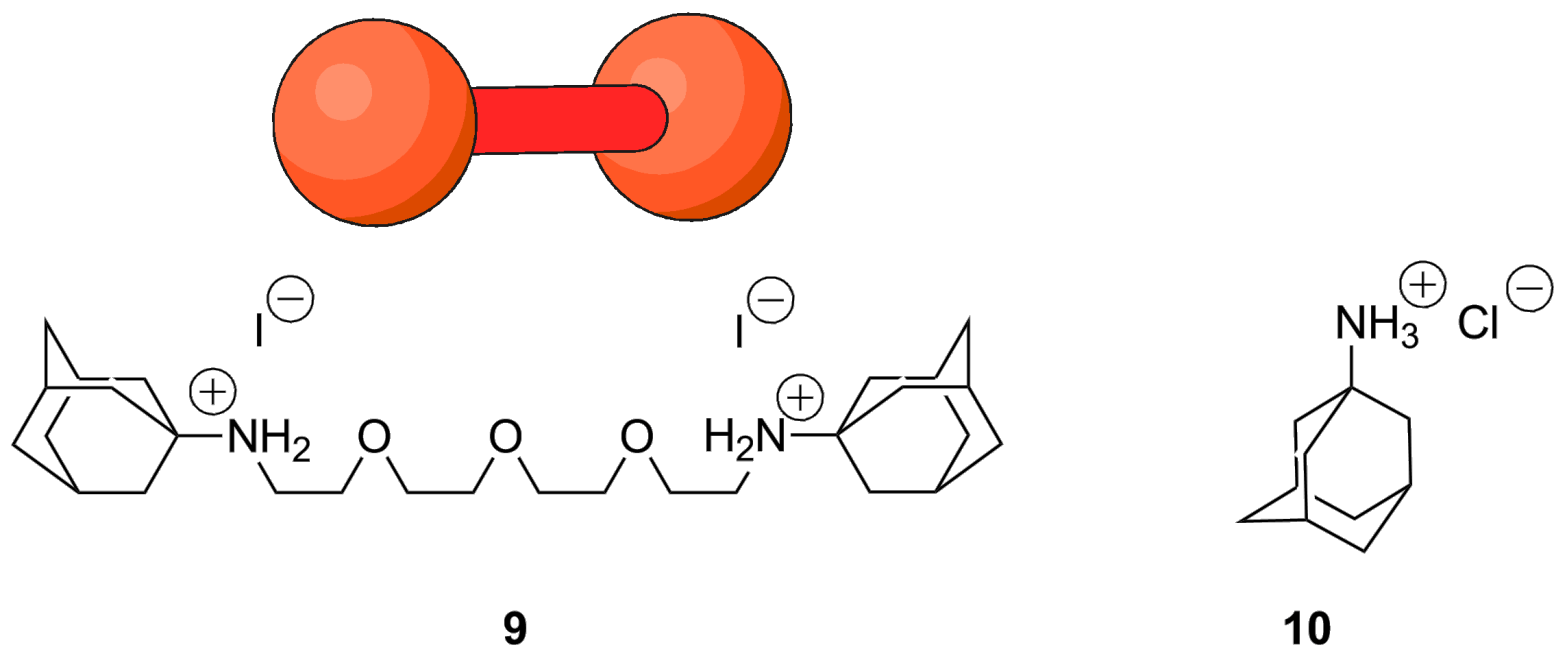
Ditopic and monotopic guest molecules.

In contrast to monomer **7**, polymer **8** was soluble in water up to a concentration of 0.15 mM (based on the repeating unit). This allows the investigation of the complexation of ditopic and monotopic guests, **9** and **10**, respectively. The solubility of the host polymer **8** as a function of the concentration of both guests **9** and **10** (Scheme 2) was determined by UV−vis spectroscopy using the extinction coefficient ε of **8** (14,800 M^−1^ cm^−1^) at 425 nm. A more detailed description of the solubility measurements is presented in Supporting Information File 1.

**Figure 6:**
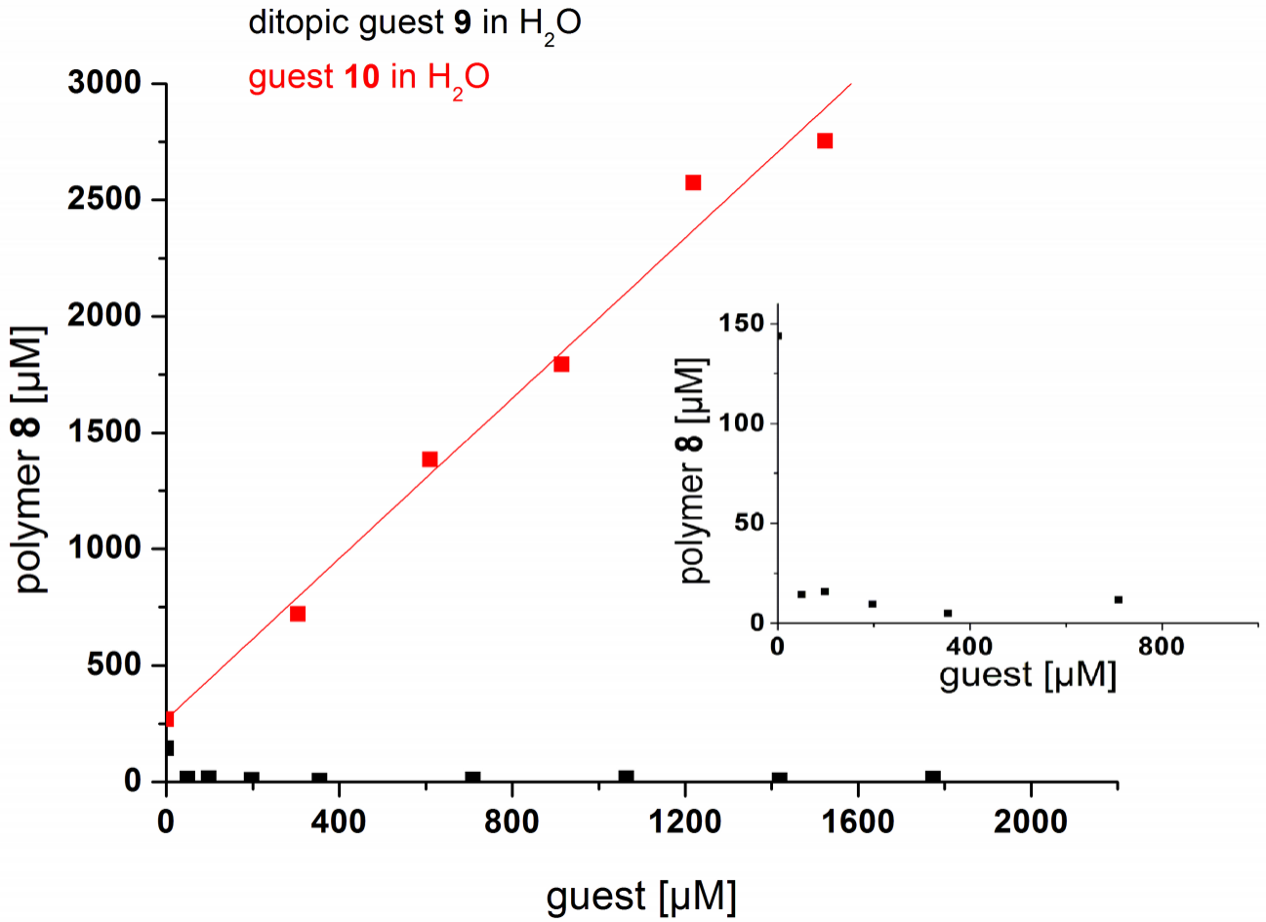
Solubility of polymer **8** in the presence of ditopic connector **9** (black graph) and 1-aminoadamantane hydrochloride **10** (red graph), respectively in water at 25 °C.

Addition of hydrophilic guest **10** caused an increase in solubility of host polymer **8** in water (Figure 6). The surprisingly steep initial slope of the phase solubility diagram, *m* = 1.4 (repeating unit/guest) could be well represented by a model where every second CD moiety has to be complexed by the hydrophilic guest to significantly improve the solubility in water. Binding constants of about 40,000 M^−1^, which were in the same range as literature values for the incorporation of adamantane derivatives into β-CD, [61] were obtained using ITC measurements considering a two-step sequential complexation with guest **10**. Further information is provided in Supporting Information File 1. Incomplete complexation with cationic guest molecules is indicated by a significant lower binding constant of 670 M^−1^ for the second binding complexation step, which is strongly inhibited as a result of the electronic repulsion of charged guest molecules in close proximity to each other. In contrast, a pronounced reduction of the solubility of CD polymer **8** was observed in the presence of ditopic guest **9**, which was attributed to the interconnection of polymer chains through the complexation of the ditopic guest. The very low concentration of connector **9** necessary for the almost complete precipitation of the host polymer **8** can be explained by the high integrability of the host–guest system based on the shape-persistence of the polyconjugated polymer backbone of **8**.

### Attachment of polymer **8** to silicon surfaces

**Scheme 3:**
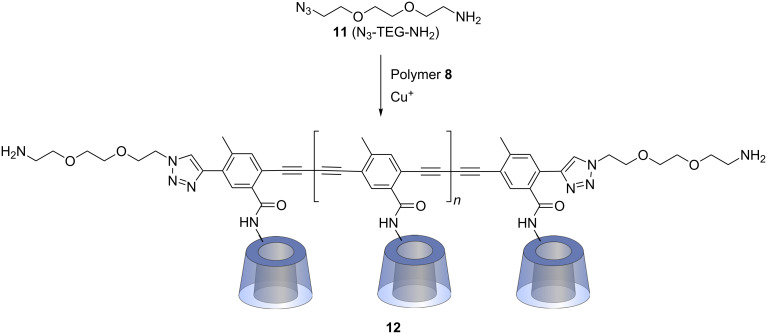
Synthesis of amino functionalized polymer **12**.

Planar silicon wafers, as well as the silicon AFM tip, were first functionalized by a polysiloxane monolayer bearing isothiocyanate groups, which smoothly react with amines forming stable thiourea links [48]. Monolayers of β-CD or β-CD-polymer were obtained by attachment of monoamino β-CD or amino-modified CD polymer **12**, synthesized from polymer **8** (Scheme 3) through Cu(I)-catalyzed azide–alkyne cycloaddition (CuAAC) with the triethylene glycol linker **11** (N_3_-TEG-NH_2_) which had been prepared in a five-step procedure [62–63].

### Probing multivalent interactions by AFM

**Figure 7:**
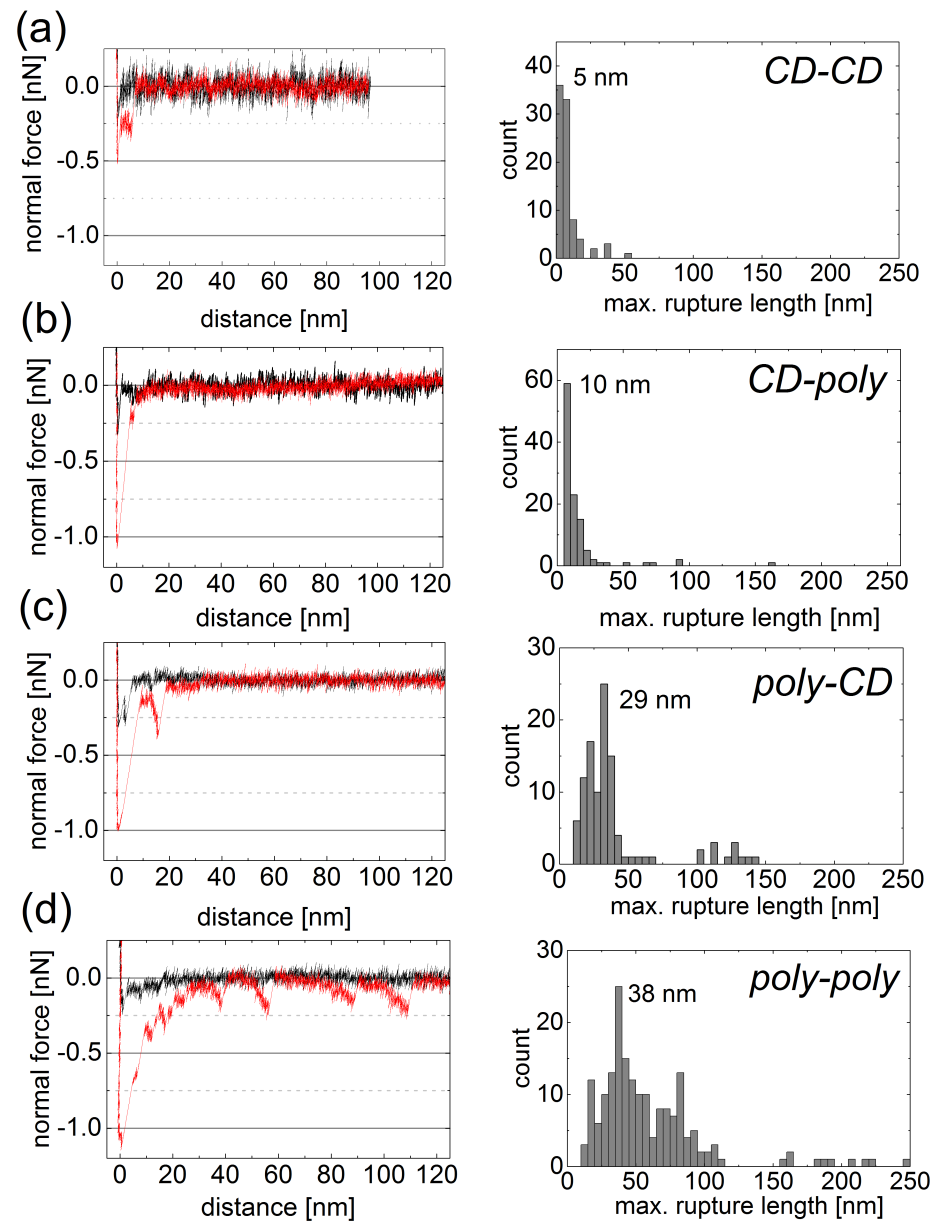
Characteristic force curves recorded during retraction of the AFM tip from the surface. Four functionalizations are compared: (a) cyclodextrin (CD) layers on tip and surface, (b) CD layer on the tip and polymers (**12**) on the surface, (c) polymers (**12**) on the tip and CD layer on the surface, and (d) polymers (**12**) on tip and surface. Black curves represent control experiments in pure water, red curves experiments in solution containing ditopic connector **9**. The maximum negative force is referred to as the pull-off force. The histograms summarize the distribution of maximum rupture length for every configuration.

The adhesive forces of **12**, due to supramolecular interactions with ditopic guest **9**, between a planar silicon surface and an AFM tip both modified with the CD polymer **12** or 6-monoamino-6-deoxy-β-CD were systematically investigated by AFM. While adhesion was very weak in pure water, significant adhesion took place over a wide range of distances in a 10 μM solution of ditopic guest **9** (Figure 7a–d). For comparison, we also investigated the adhesion forces between CD and **12**, **12** and CD, and CD and CD at the tip and the planar surface, respectively, caused by the adamantane connector **9**. Adhesive forces were recorded as function of the tip–surface distance upon retracting of the tip from the surface for all four configurations. The pull-off force required to detach the tip from the surface in the presence of connector molecules was of the order of 500 pN for the CD–CD configuration and about 1 nN for all configurations involving CD polymers (**12**). These values are significantly higher than the pull-off forces of about 250 pN measured in control experiments for all configurations. The graphical summary in Figure 7a suggests that the pull-off forces for the **12**–**12** configuration are slightly higher than for the **12–**CD and for the CD**–12** configuration.

While the pull-off force is similar, the overall appearance of the force curves differs for the three polymer configurations. The interaction distance varies significantly for the different configurations. The CD–CD configuration has the shortest and the polymer–polymer configuration the longest range of interactions. The interaction range can be quantified by the tip–surface distance at which the last rupture occurs, referred to as maximum rupture length. The histograms of the maximum rupture length for all four configurations are presented in Figure 7. For the CD–CD configuration, the most probable maximum rupture length of 5 nm corresponds to the combined height of the monolayers on tip and surface, each of about 2.5 nm. The typical rupture length for the CD–**12** configuration is 10 nm, while it is 29 nm for the **12**–CD configuration. The difference in maximum rupture length indicates a difference in the detachment mechanism. In the CD–**12** configuration, the polymers bind to the sloped facets of the asperity of the AFM tip. Upon pulling, the polymers are sheared from the tip apex by rupturing all bonds simultaneously leading to one large rupture peak at a small tip–surface distance. For the **12**–CD configuration, a force plateau observed in the force-distance curve in Figure 7c reveals the peeling of a polymer chain from the CD-coated surface resulting in a rupture length similar to the length of the polymer chains.

For the **12**–**12** configuration, many additional small detachment events lead to a broadening of the pull-off curve and reveal the rupture of bonds for tip–surface distances as large as 110 nm in Figure 7d. The broad distribution of rupture length, which extends to roughly the double of that of the **12**–CD configuration, indicates that individual long polymer chains interlock, explaining also the characteristic stretching events in the force-distance curve. The most probable maximum rupture length for the **12**–**12** system is 38 nm, which is double of the average polymer length of 17 nm predicted from the MALDI–TOF and SANS/SAXS results. The agreement confirms the picture that the maximum rupture length reflects the final detachment of supramolecular bonds at the end of stretched polymer chains attached to AFM tip and surface.

The higher sensitivity of our AFM set up compared to the MALDI–TOF instrument allowed us to even detect single rupture events at a distance up to 250 nm, which proved that some individual chains had a length of at least 125 nm. Compared to MALDI–TOF measurements in which the small number of high molecular weight polymer chains are hardly detectable, AFM experiments overemphasize the few longest polymer chains probing the interactions of the regularly spaced CDs in CD polymer **12** and ditopic connector molecules. Due to this observation AFM is an excellent detection tool for analysing cooperative effects in ordered supramolecular systems.

**Figure 8:**
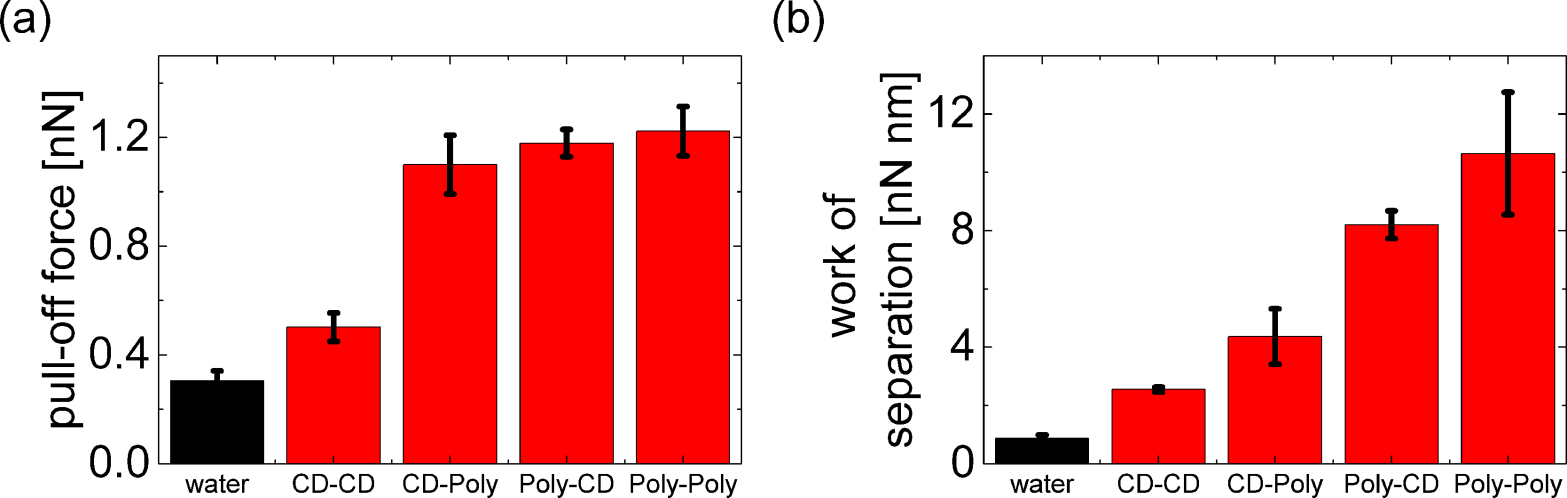
Graphical summary of experimental results for the four configurations of CD attachment introduced in Figure 2: (a) pull-off forces, (b) work of separation. The error bars indicate standard deviations for averages over different lateral positions on the functionalized surface.

The differences between the four configurations of functionalization can be further quantified by integration of the force curves, resulting in the work of separation which has been employed before as a suitable parameter for the quantification of polymer detachment [45]. In line with the characteristic shape of the example force curves, the work of separation increased significantly in the order CD–CD, CD–**12**, **12**–CD and **12**–**12** configuration (Figure 8). The relative increase in the work of adhesion from control experiments to measurements of the specific interactions caused by the connector molecule **9** was even higher than the respective increase in pull-off force due to the very short range of the non-specific adhesive interactions.

The significant difference in the interaction range and thus in the work of separation between CD–**12** and **12**–CD configuration can be explained by the asymmetry between curved tip and flat surface and the resulting difference in the detachment mechanism. Polymers attached to the surface bind to the side faces of the tip with its nanometer-scale apex radius. Upon retraction, the force acts along the polymer and shears the polymer off the tip, with all bonds rupturing more or less simultaneously. In contrast, polymers attached to the tip bind to the flat surface such that upon retraction the polymer is peeled from the surface by the orthogonal force, one bond breaking after another. The different detachment scenarios are depicted in the schematic drawings in Figure 2. The shearing configuration (CD–**12**) leads to simultaneous rupture of all bonds, while the peeling configuration (**12**–CD) involves bending of the polymer and consecutive rupture. The strongest adhesion is offered by the supramolecular interlocking of polymers attached to tip and surface. Supramolecular interconnection between two CD polymer **12** molecules through the ditopic guest **9** is expected to be superior to the one between CD polymer **12** and CD because of the higher regularity of the CD spacing at the polymer compared to the spacing within the CD monolayer. We conclude that the regularity of the CD polymer **12** allows to establish a much higher number of supramolecular bonds with the connector **9** giving rise to about a fivefold enhancement of the work of separation.

**Figure 9:**
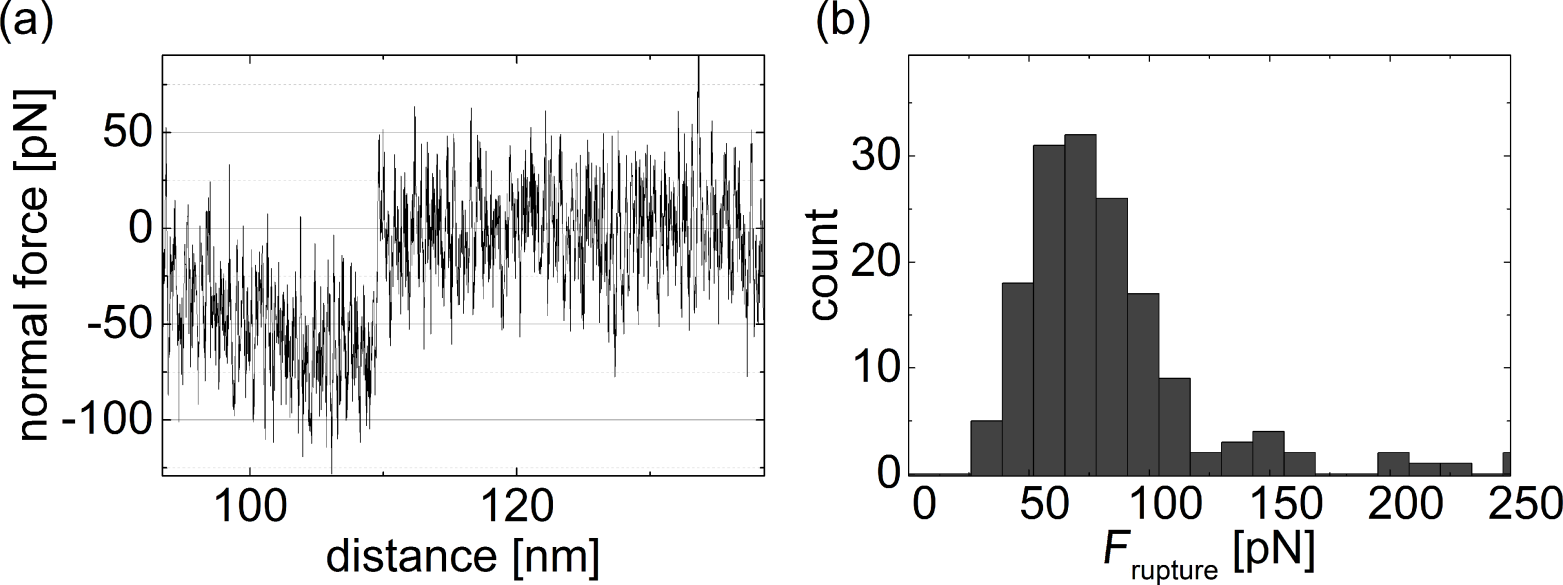
(a) Detail of the end of a force curve for a polymer-functionalized tip retracted from a polymer-functionalized surface. This is the last unbinding event which occurs at a tip–surface distance of about 110 nm, indicating the possible range of interaction for polymer functionalization of tip and surface. (b) Distribution of force drops for the last unbinding event with a maximum at 63 pN and a possible second maximum at about double this value.

Many force curves exhibit a well-defined last rupture event. A representative example is shown in Figure 9a, where the force drops from around 63 pN to zero at a distance of 110 nm. The distribution of rupture forces for the last rupture events, shown in Figure 9b, has a clear maximum at 63 pN, determined by a Gaussian fit to the distribution, and a weak second maximum at about double this value.

We conclude that 63 ± 10 pN is the rupture force for a single bond between our supramolecular polymers **12** established by the ditopic guest **9**. The value agrees with rupture force measured for adamantane–CD complexes with CD molecules in the surface layers when the stiffness of the AFM cantilever is taken into account [64].

Force curves like those shown in Figure 7 can be repeated on the same spot of one sample many times with very similar results. The repeatability confirms the reversibility of the underlying interactions. It is difficult to estimate the number of supramolecular bonds contributing to pull-off forces of 1 nN in Figure 7. Based on the single bond rupture force one could assume contributions by 16 supramolecular bonds or even more since it is unlikely that all bonds are loaded to their rupture force. As long as we have no experimental means to exactly determine the number of polymers molecules involved we cannot evaluate the number of interconnections per polymer. Since a significant number of single rupture events at large tip–surface distances require forces of around 200–250 pN (Figure 7d) up to four bonds per polymer pair appear reasonable.

### Probing multivalent interactions by friction AFM

**Figure 10:**
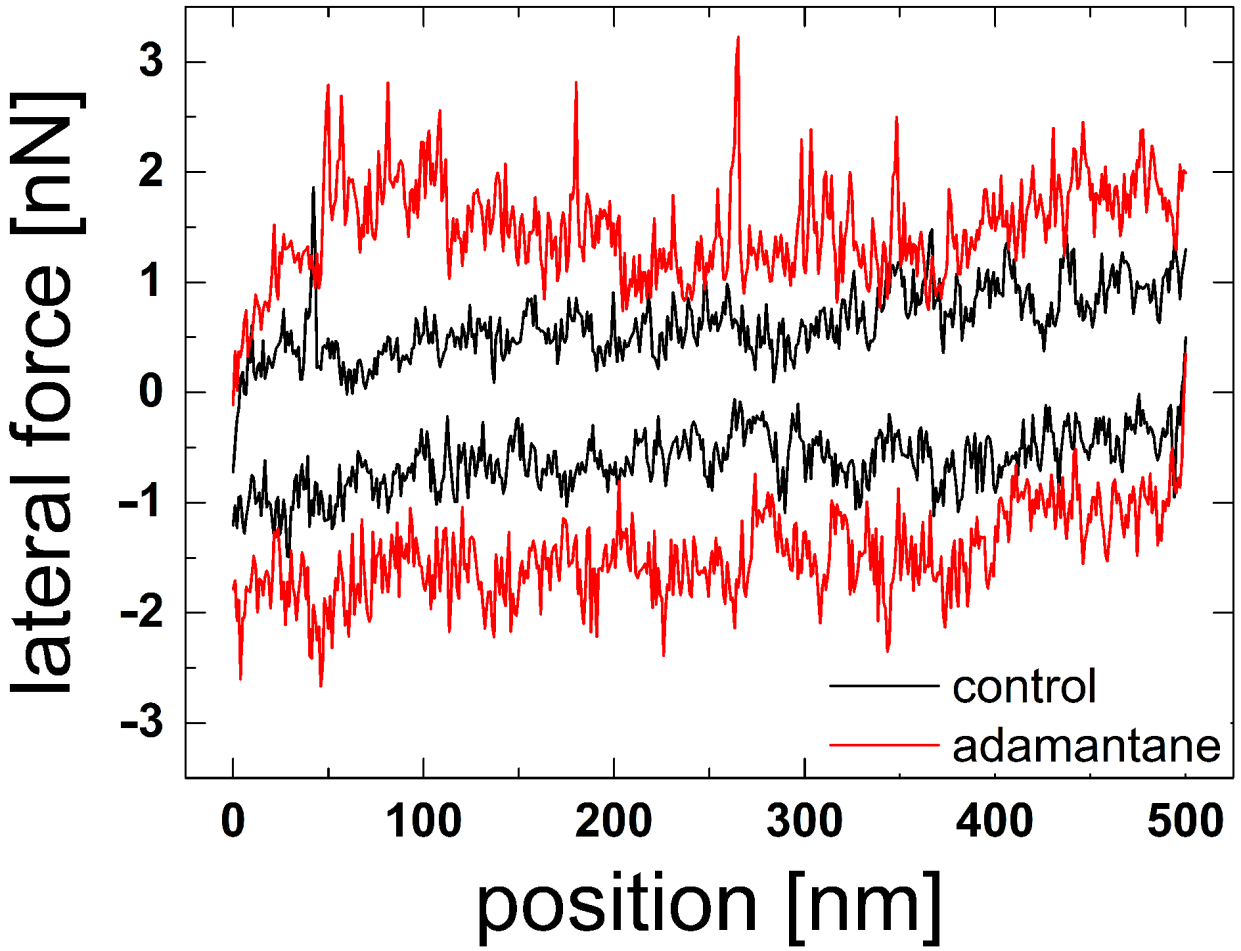
Characteristic result of a friction experiment for a polymer-functionalized tip sliding on a surface carrying a CD layer. Lateral forces are plotted as a function of lateral tip position when sliding 500 nm back and forth with a velocity of 45 nm/s.

Finally, friction force experiments have been performed for the **12**–CD configuration. The tip of the AFM slides in contact across the surface, where polymers attached to the tip may interact with the CD layer on the surface. A characteristic result is presented in Figure 10. The average friction force increases by a factor of 2.5 due to the supramolecular interactions in comparison with control experiments in water. The friction force curve exhibit peculiar spikes when adamantane connector molecules are present. These spikes represent an irregular stick-slip motion of the tip. When one or several polymers are bound to the surface, the tip is stuck and the increasing force leads to torsion of the cantilever until the force is large enough to detach the polymers and drag them further across the surface. The highest friction force spikes of the **12**–CD configuration exhibit a force drop of 2 nN, similar to the highest pull-off forces for the same system. Shearing of a series of bonds, as described for adhesion in the CD–**12** configuration, is also the mechanism underlying friction in the **12**–CD configuration. Stick spike forces of 2 nN are enhanced by at least a factor of 3 compared to the one for the CD–CD system previously described [48]. This spike force may be enhanced by the multivalency effects discussed above, but its strength indicates that more than one polymer molecule might be involved.

## Conclusion

In conclusion, regular water-soluble shape-persistent CD polymers based on poly(phenylene butadiynylene) were prepared by a straightforward Glaser coupling/click chemistry approach, which can be attached to planar silicon surfaces as well as AFM tips. Structural perfection of the resulted polymers was confirmed by MALDI–TOF measurements revealing the presence of high molecular weight materials with up to 38 repeat units. High integrability of the scaffold was proven by UV–vis supported solubility measurements upon addition of ditopic adamantane connectors. Small-angle neutron scattering and X-ray experiments reveal the presence of stiff cylindrical polymer chains with contour lengths of about 13–16 nm, which corresponds to the values obtained by MALDI and AFM measurements. Hard substrates with the shape-persistent polymers and interconnected by ditopic guest molecules require about five times higher separation energies than those functionalized with conventional CD monolayers. This significant enhancement of adhesion can be attributed to a strong cooperative effect favored by the rigidity of the polymer backbone and the regular spacing of the CD moieties. The range of adhesive interactions could be extended from 5 to 38 nm, which will also allow the interconnection of surfaces with higher roughness. The stiff polymers exhibit a clear contrast between shearing and peeling mechanisms, depending on the geometrical configuration of attachment. The distribution of the maximum rupture lengths in the force microscopy experiments confirms the molecular weight distribution of the CD polymers estimated by MALDI–TOF and the average contour length determined by SANS/SAXS. In addition, force microscopy experiments emphasize the longest polymer chains and their maximum length.

## Supporting Information

File 1 Experimental procedures, MALDI–TOF spectra, details on SANS/SAXS instrumentation and analysis, surface preparation protocols and other instrumentation parameters.
