# Supplementary material for: Interactions between shape-persistent macromolecules as probed by AFM
Source: Beilstein J Org Chem. 2017 May 18;13:938–51. doi: 10.3762/bjoc.13.95 (PMC5480325; doi:10.3762/bjoc.13.95)
Supplement: File 1 — Experimental procedures, MALDI–TOF spectra, details on SANS/SAXS instrumentation and analysis, surface preparation protocols and other instrumentation parameters. [file Beilstein_J_Org_Chem-13-938-s001.pdf]

Supporting Information

for

Interactions between shape-persistent macromolecules as probed by AFM

Johanna Blass[‡1,2], Jessica Brunke,[‡3] Franziska Emmerich[1,2], Cédric Przybylski[4], Vasil M. Garamus[5], Artem Feoktystov[6], Roland Bennewitz[1,2], Gerhard Wenz[3] and Marcel Albrecht[*3]

Address: [1]INM-Leibniz-Institute for New Materials, Saarland University, Campus D 2.2, D-66123 Saarbrücken, Germany, [2]Physics Department, Saarland University, Campus D 2.2, D-66123 Saarbrücken, Germany, [3]Organic Macromolecular Chemistry, Saarland University, Campus C 4.2, D-66123 Saarbrücken, Germany, [4]UPMC, IPCM-CNRS UMR 8232, Sorbonne Universités, 75252 Paris Cedex 05, France [5]Helmholtz-Zentrum Geesthacht (HZG), Centre for Materials and Costal Research, Max-Planck-Str. 1, 21502 Geesthacht, Germany, [6]Jülich Centre for Neutron Science (JCNS) at Heinz Maier-Leibnitz Zentrum (MLZ), Forschungszentrum Jülich GmbH, Lichtenbergstr. 1, 85748 Garching, Germany

Email: Marcel Albrecht[*] - m.albrecht@mx.uni-saarland.de

*Corresponding author

‡Equal contributors

Experimental procedures, MALDI–TOF spectra, details on SANS/SAXS instrumentation and analysis, surface preparation protocols and other instrumentation parameters

Experimental Section

1. Synthetic Protocols

1,4-Dibromo-2,5-dimethylbenzene (1)

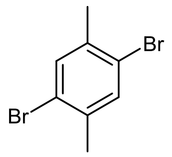

1

1,4-Dibromo-2,5-dimethylbenzene (**1**) was synthesized according to a known procedure starting from *p*-xylene and bromine with a catalytic amount of iodine in 87 % yield [1].

2,5-Dibromo-4-methylbenzoic acid (2)

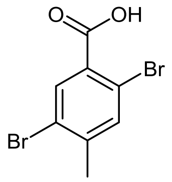

2

Selective oxidation of one methyl group was obtained by reaction of **1** with nitric acid resulting in the corresponding benzoic acid derivative (**2**) (42 % yield) [2].

tert-Butyl 2,5-dibromo-4-methylbenzoate (3)

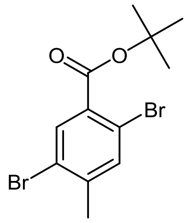

3

Magnesium sulfate (15.00 g, pre-dried in vacuo) was suspended in dry dichloromethane (50 mL). Concentrated sulfuric acid (1.60 mL, 30 mmol) was added and the mixture was stirred at rt for 15 min. After addition of **2** (8.82 g, 30 mmol) and *tert*-butanol (14.30 mL, 150 mmol) the tightly stoppered mixture was stirred at rt for 4 d. The resulting suspension was quenched with saturated bicarbonate solution and the aqueous phase was extracted with dichloromethane (3 × 100 mL). Drying over magnesium sulfate and purification by column chromatography (SiO_2, *n*-hexane/dichloromethane 6:1) resulted in product **3** (7.77 g, 72%) as a light yellow oil.

^{1}H NMR: (400 MHz, $CDCl_3$) δ = 7.88 (s, 1H, H_{Phenyl}), 7.50 (s, 1H, H_{Phenyl}), 2.40 (s, 3H H_{Methyl}), 1.61 (s, 9H, $H_{tert\text{-}Butyl}$) ppm.

^{13}CNMR: (100.6 MHz, $CDCl_3$) δ = 164.1 (C), 142.5 (C), 135.8 (CH), 134.5 (CH), 132.7 (C), 123.3 (C), 119.8 (C), 82.9 (C), 28.1 (CH_3), 22.6 (CH_3) ppm.

HRMS: calculated for $C_{12}H_{14}O_2Br_2$ 347.9361

found 347.9371.

***tert*-Butyl 4-methyl-2,5-bis((trimethylsilyl)ethynyl)benzoate (4)**

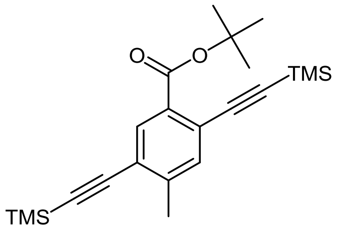

4

Palladium(II) chloride (222 mg, 1.25 mmol), copper(I) iodide (119 mg, 0.625 mmol) and triphenylphosphine (1.48 g, 5.63 mmol) were stirred at reflux temperature for 20 min. After cooling at rt **3** (4.35 g, 12.50 mmol) was added and the resulting mixture

was stirred at rt for 10 min. Trimethylsilyl acetylene (5.30 mL, 37.50 mmol) was added and the reaction mixture was stirred at 80 °C for 48 h. The solvent was evaporated under reduced pressure and the resulting solid was suspended in *n*-pentane. After filtration over celite and washing with *n*-pentane the filtrate was evaporated. The crude mixture was purified by column chromatography (SiO_2, *n*-hexane/dichloromethane 6:1) yielded product **4** (2.98 g, 62%) as a light yellow oil.

^{1}H NMR: (400 MHz, $CDCl_3$) δ = 7.85 (s, 1H, H_{Phenyl}), 7.39 (s, 1H, H_{Phenyl}), 2.41 (s, 3H H_{Methyl}), 1.61 (s, 9H, $H_{tert\text{-}butyl}$), 0.26 (s, 18H, H_{TMS}) ppm.

^{13}CNMR: (100.6 MHz, $CDCl_3$) δ = 165.2 (C), 143.2 (C), 135.4 (CH), 133.6 (CH), 132.0 (C), 123.1 (C), 122.2 (C), 103.5 (C), 102.6 (C), 100.9 (C), 100.3 (C), 81.9 (C), 28.2 (CH_3), 20.3 (CH_3), -0.10 (CH_3) ppm.

HRMS: calculated for $C_{22}H_{32}O_2Si_2$ 384.1941

found 384.1938.

***tert*-Butyl 2,5-diethynyl-4-methylbenzoate (5)**

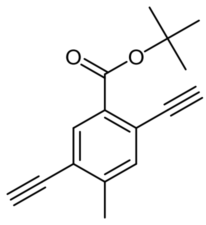

5

TMS-protected *tert*-butyl ester **4** (2.98 g, 7.75 mmol) was dissolved in dry THF (100 mL). After cooling to −20 °C tetra-*n*-butylammonium fluoride (1 M in THF, 16.25 mL, 16.25 mmol) was added dropwise and stirring was continued at −20 °C for 30 min. The mixture was quenched with saturated ammonium chloride solution and extracted with dichloromethane (3 × 100 mL). After drying over magnesium sulfate

and purification by column chromatography (SiO_2, *n*-hexane/dichloromethane 5:2) product **5** (1.62 g, 87%) was isolated as a light brown solid.

^{1}H NMR: (400 MHz, $CDCl_3$) δ = 7.98 (s, 1H, H_{Phenyl}), 7.44 (s, 1H, H_{Phenyl}), 3.43 (s, 1H, $H_{Acetylene}$), 3.39 (s, 1H, $H_{Acetylene}$), 2.45 (s, 3H, H_{Methyl}), 1.60 (s, 9H, $H_{tert\text{-}Butyl}$) ppm.

^{13}CNMR: (100.6 MHz, $CDCl_3$) δ = 164.4 (C), 144.0 (C), 135.8 (CH), 134.4 (CH), 131.6 (C), 122.4 (C), 122.3 (C), 83.2 (CH), 83.2 (CH), 82.2 (C), 82.1 (C), 81.2 (C), 28.1 (CH_3), 20.3 (CH_3) ppm.

HRMS: calculated for $C_{16}H_{16}O_2$ 240.1150

found 240.1129.

2,5-Diethynyl-4-methylbenzoic acid (6)

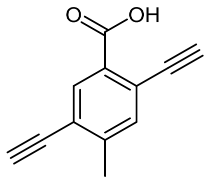

6

tert-Butyl ester **5** (3.13 g, 13.00 mmol) was dissolved in dry dichloromethane (100 mL). After addition of trifluoroacetic acid (19.20 mL, 250 mmol) the mixture was stirred at rt for 18 h. The solvent was evaporated under reduced pressure and the crude product was purified by column chromatography (SiO_2, dichloromethane/methanol 100:1) yielded **6** (1.41 g, 60%) as a light yellow solid.

^{1}H NMR: (400 MHz, DMSO-d_6) δ = 13.18 (bs, 1H, H_{COOH}), 7.85 (s, 1H, H_{Phenyl}), 7.54 (s, 1H, H_{Phenyl}), 4.61 (s, 1H, $H_{Acetylene}$), 4.48 (s, 1H, $H_{Acetylene}$), 2.40 (s, 3H, H_{Methyl}) ppm.

^{13}CNMR: (100.6 MHz, DMSO-d_6) δ = 158.9 (C), 136.1 (C), 127.3 (CH), 125.8 (CH), 122.6 (C), 114.6 (C), 114.4 (C), 75.9 (CH), 75.8 (CH), 73.1 (C), 72.2 (C), 11.0 (CH_3) ppm.

HRMS: calculated for $C_{12}H_8O_2$ 184.0524

found 184.0522.

Monomer 7

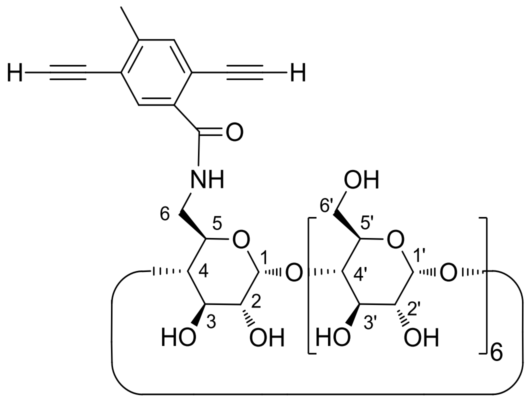

7

Applying the synthesis approach of Ritter et al.[3] the CD-modified monomer **7** was prepared by amide coupling reaction of benzoic acid **6** and monoamino-modified β-CD.

6-Monoamino-6-deoxy-β-CD [5] (567 mg, 0.50 mmol) was dissolved in dry DMF (20 mL). After cooling to 0 °C benzoic acid derivative **6** (120 mg, 0.65 mmol), *N,N'*-dicyclohexylcarbodiimide (165 mg, 0.80 mmol) and 1-hydroxybenzotiazole (107 mg, 0.70 mmol) were added and the resulting mixture was stirred at rt for 8 d. The resulting suspension was filtrated and the filtrate was poured into a large excess of acetone. The precipitate was collected and suspended in distd water. After filtration and washing with distd water product **7** (352 mg, 54%) was obtained as a white solid.

^{1}H NMR: (400 MHz, DMSO-d$_6$) δ = 8.19 (m, 1H, H_{NH}), 7.57 (s, 1H, H_{Phenyl}), 7.47 (s, 1H, H_{Phenyl}), 5.79-5.66 (m, 14H, $H_{Hydroxy}$), 4.92 (m, 1H, $H_{CH-1'}$), 4.83 (m, 6H, H_{CH-1}), 4.54 (s, 1H, $H_{Acetylene}$), 4.43 (m, 6H, $H_{Hydroxy}$), 4.36 (s, 1H, $H_{Acetylene}$), 3.75-3.36 (m, 35H, H_{CH-3}, H_{CH-5}, H_{CH2-6}), 3.35-3.30 (m, 14H, H_{CH-2}, H_{CH-4}), 2.39 (s, 3H, H_{Methyl}) ppm.

MALDI-TOF: calculated for $[M+Na]^+$ 1322.42

found 1322.42.

calculated for $[M+K]^+$ 1338.39

found 1338.28.

Polymer 8

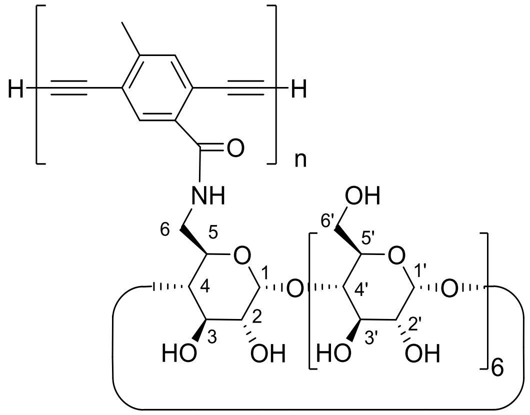

8

CD-modified monomer **7** (150 mg, 0.12 mmol) was dissolved in dry pyridine (4 mL). After addition of copper(I) chloride (9 mg, 0.09 mmol) and copper(II) acetate hydrate (22 mg, 0.12 mmol) the reaction mixture was stirred at 60 °C for 24 h. The solvent was evaporated under reduced pressure and the resulting orange colored solid was purified using ultrafiltration (VIVASPIN TURBO 4, 5000 Da) in water/pyridine 10:1, (v/v). Product polymer **8** was obtained as an orange colored solid (136 mg, 91%).

^1H NMR: (400 MHz, DMSO-d_6) δ = 8.00-7.75 (m, 2H, H_{Phenyl}), 5.85-5.67 (m, 14H, $H_{Hydroxy}$), 4.84 (m, 7H, H-1), 4.45 (m, 6H, $H_{Hydroxy}$), 3.63 (m, 35H, H_{CH-3}, H_{CH-5}, H_{CH2-6}), 3.32 (m, 14H, H_{CH-2}, H_{CH-4}), 2.55 (m, 3H, H_{Methyl}) ppm.

Polymer 12

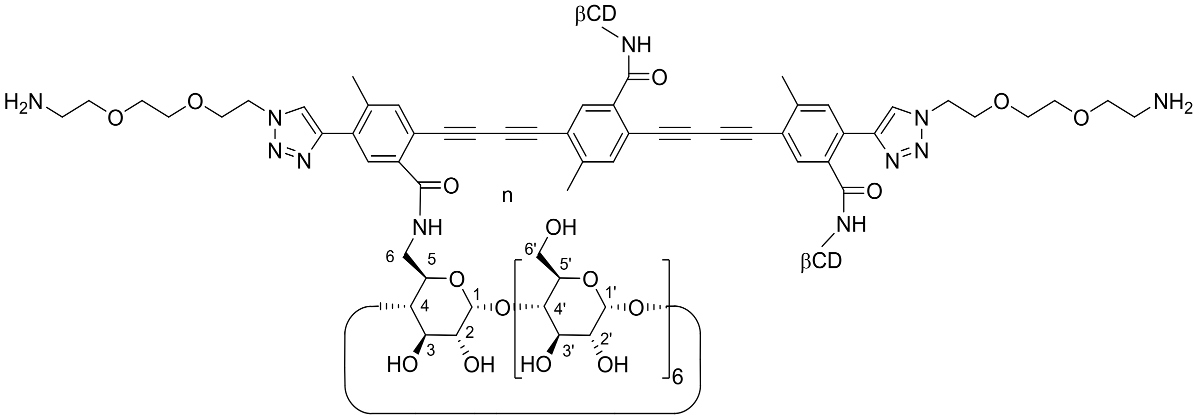

12

CD-modified polymer **8** (30 mg, 0.023 mmol) and triethylene glycol linker **11** (N_3-TEG-NH_2) (16 mg, 0.092 mmol) were dissolved in DMSO (2 mL). After addition of copper(II) sulfate pentahydrate (1 mg, 0.004 mmol) and sodium ascorbate (2 mg, 0.010 mmol) in 0.2 ml distd water the reaction mixture was stirred at rt for 2 d. The mixture was lyophilized and purified using ultrafiltration (VIVASPIN TURBO 4, 5000 Da). Product polymer **12** was obtained as an orange colored solid (25 mg, 83%).

^1H NMR: (400 MHz, DMSO-d_6) δ = 8.02-7.88 (m, 2H, H_{Phenyl}), 5.90-5.58 (m, 14H, $H_{Hydroxy}$), 4.84 (m, 7H, H-1), 4.46 (m, 6H, $H_{Hydroxy}$), 3.64 (m, 35H, H_{CH-3}, H_{CH-5}, H_{CH2-6}), 3.32 (m, 14H, H_{CH-2}, H_{CH-4}), 2.55 (m, 3H, H_{Methyl}) ppm. Proton signals of the end groups could not be detected.

2. Mass spectrometry

MALDI–TOF MS experiments were performed using a Perseptive Biosystems Voyager-DE Pro STR MALDI–TOF mass spectrometer (Applied Biosystems/MDS Sciex, Foster City, CA) which was equipped with a nitrogen UV laser (λ = 337 nm) pulsed at a 20 Hz frequency. The mass spectrometer was operated in the positive ion linear mode with an accelerating potential of +25 kV and a grid percentage equal to 93%. Mass spectra were recorded with the laser intensity set just above the ionization threshold (2800–3200 in arbitrary units, on our instrument) to avoid fragmentation, maximizing the resolution (pulse width 3 ns) and to result in a strong analyte signal with minimal matrix interference. The time delay between laser pulse and ion extraction was set to 800 ns. Typically, mass spectra were obtained by accumulation of 600–1000 laser shots, and further processed using Data Explorer 4.0 software (Applied Biosystems).

Sample preparation was performed in a mixture of acetonitrile/water 50:50 (v/v) with a concentration of 5 mg/mL. Matrix incorporation of the analyte was conducted by mixing 0.5 to 1 µL of sample and one volume 1:1 (v/v) of matrix. One microliter of the mixture was deposited on a mirror polished stainless steel MALDI target and allowed to dry at room temperature under atmospheric pressure over a period of five minutes. External calibration was performed using a protein mixture provided by the manufacturer.

The HABA/TMG_2, ionic liquid matrix was prepared as previously described [5]. Briefly, 2-(4’-hydroxyphenylazo)benzoic acid (HABA) was mixed with 1,1,3,3-tetramethylguanidine (TMG) at a 1:2 molar ratio in aceonitrile, and the obtained solution was then sonicated for 15 min at 40 °C. After removal of the acetonitrile by centrifugal evaporation in a SpeedVac for 3 h at room temperature, ILM was left in

vacuum overnight. ILM was then prepared at a concentration of 90 mg/mL in acetonitrile.

Polymer materials were analyzed by different average values to determine molecular weight information such as mass average molecular weight (M_w), the number average molecular weight (M_n) and the resulting polydispersity index (PDI = M_w / M_n).

$$M_n = \frac{\sum_i N_i M_i}{\sum_i N_i} \ (1); \ M_w = \frac{\sum_i N_i M_i^2}{\sum_i N_i M_i} \ (2); \quad \mathrm{PDI} = \frac{M_w}{M_n} \ (3)$$

Such values can be approximate by MALDI-TOF mass spectrometry considering N_i as the relative abundance (%) and M_i as the measured *m*/*z* (see Table S1). Applying aforementioned formula, to the two fractions, led to $\overline{M_n}$ and $\overline{M_w}$ values.

MALDI spectrum of polymer 8 (soluble fraction)

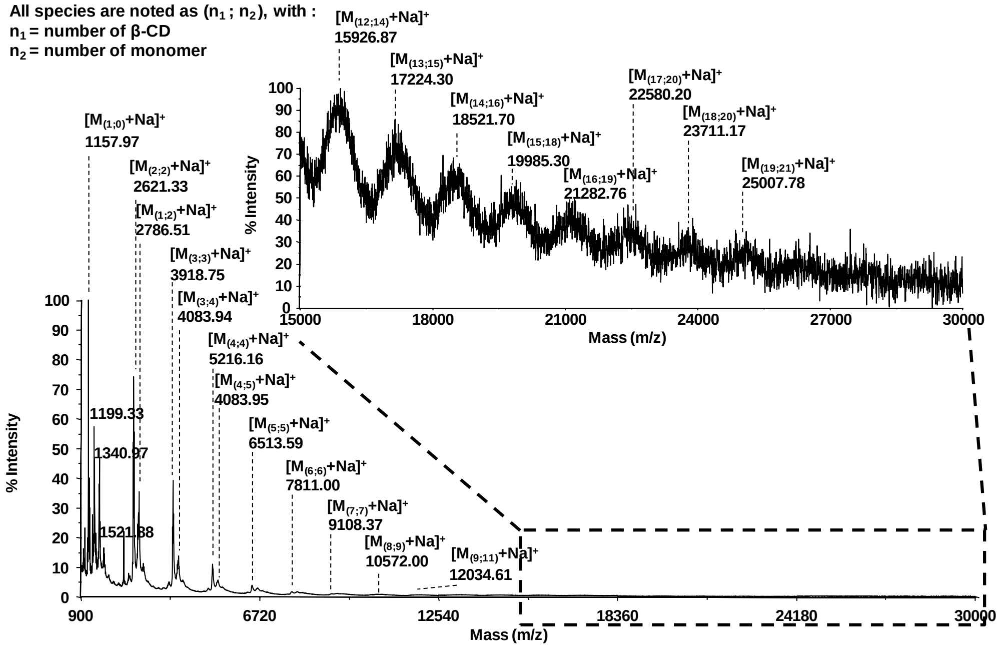

Table S1: Characteristics of the most intense ions according to detected population in the soluble fraction (acetonitrile/water 50/50) of compound **8** sample. Only those signals with a signal to noise ratio ≥ 3 were reported.

Mass (m/z) as $[M+Na]^+$		Mass accuracy (ppm)	Relative abundance (%)	Putative assignment	
Exp.	Theo.			Number of β-CD	Number of Benzoic acid derivative
2621.33	2621.32	2	55.7	2	2
3918.76	3918.74	6	29.4	3	3
5216.16	5216.15	2	6.9	4	4
6513.59	6513.56	5	2.0	5	5
7811.00	7810.97	4	1.2	6	6
9108.37	9108.38	-2	0.8	7	7
10572.00	10572.25	-24	0.5	8	9
12034.61	12034.86	-21	0.5	9	12
13332.01	13332.27	-19	0.5	10	13
15926.87	15927.09	-14	0.4	12	14
17224.30	17224.51	-12	0.3	13	15
18521.70	18521.92	-11	0.3	14	16
19985.30	19986.01	-36	0.3	15	18
21282.76	21283.42	-31	0.3	16	19
22580.20	22580.84	-28	0.3	17	20
23711.17	23711.57	-17	0.3	18	20
25007.78	25008.98	-48	0.3	19	21

MALDI spectrum of polymer 8 (insoluble fraction)

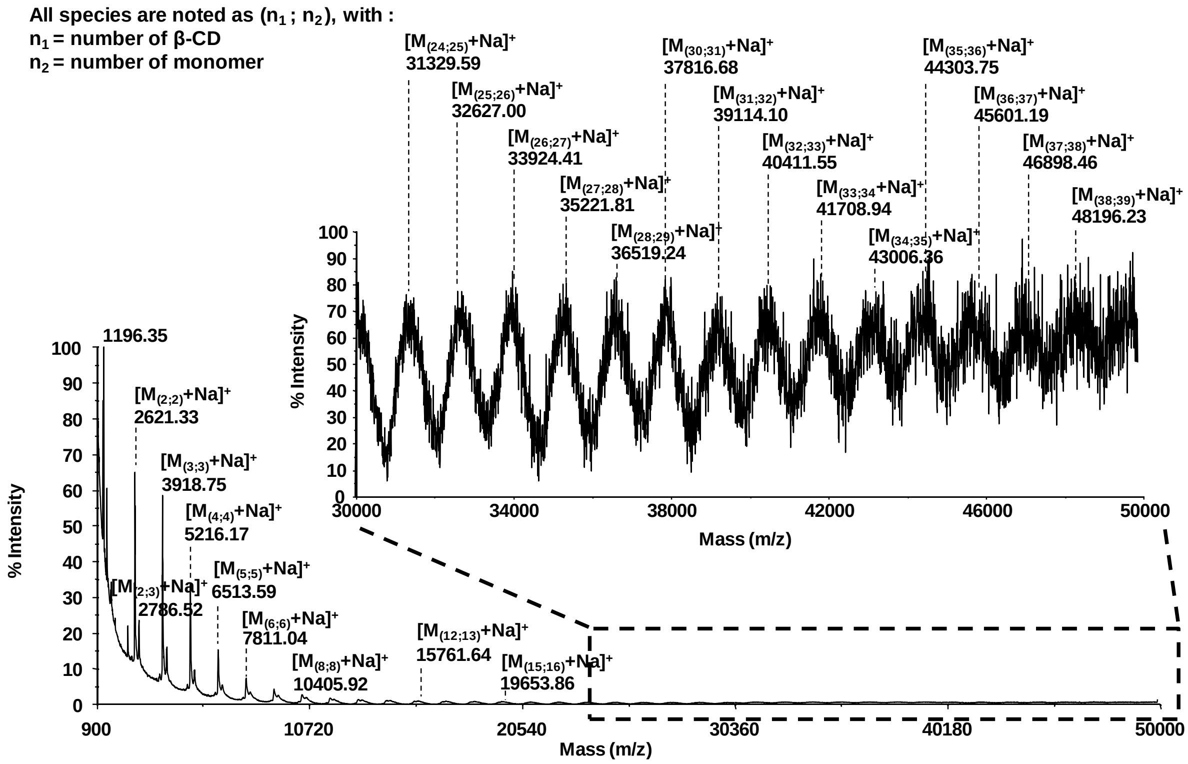

Table S2: Characteristics of the most intense ions according to the detected population in the insoluble fraction (acetonitrile/water 50:50) of compound **8** sample. Only those with signal to noise ratio ≥ 3 were reported.

Mass (*m/z*) as [M+Na]$^+$		Mass accuracy (ppm)	Relative abundance (%)	Putative assignment	
Exp.	Theo.			Number of β-CD	Number of Benzoic acid derivative
2621.33	2621.32	2	28.5	2	2
3918.75	3918.74	4	25.6	3	3
5216.17	5216.15	4	14.5	4	4
6513.59	6513.56	5	6.7	5	5
7811.04	7810.97	9	3.4	6	6
9108.42	9108.38	4	1.9	7	7
10405.92	10405.79	12	1.3	8	8
11703.32	11703.21	10	1.1	9	9
13000.58	13000.62	-3	0.9	10	10

14297.91	14298.03	-8	0.8	11	11
15761.64	15761.90	-16	0.7	12	13
17059.21	17059.31	-6	0.7	13	14
18356.78	18356.73	3	0.6	14	15
19653.86	19654.14	-14	0.7	15	16
20951.10	20951.55	-21	0.4	16	17
22248.86	22248.96	-5	0.6	17	18
23546.40	23546.37	1	0.6	18	19
24843.25	24843.79	-22	0.6	19	20
26141.15	26141.20	-2	0.6	20	21
27438.17	27438.61	-16	0.6	21	22
28736.10	28736.02	3	0.6	22	23
30033.03	30033.43	-13	0.6	23	24
31329.59	31330.85	-40	0.6	24	25
32627.00	32628.26	-39	0.6	25	26
33924.41	33925.67	-37	0.6	26	27
35221.81	35223.08	-36	0.6	27	28
36519.67	36520.49	-23	0.6	28	29
37816.68	37817.91	-32	0.6	29	30
39114.10	39115.32	-31	0.6	30	31
40411.55	40412.73	-29	0.6	31	32
41708.94	41710.14	-29	0.6	32	33
43006.96	43007.55	-14	0.6	33	34
44303.75	44304.97	-27	0.6	34	35
45601.19	45602.38	-26	0.6	35	36
46898.46	46899.79	-28	0.6	36	37
48196.23	48197.20	-20	0.6	37	38

MALDI spectrum of monomer 7

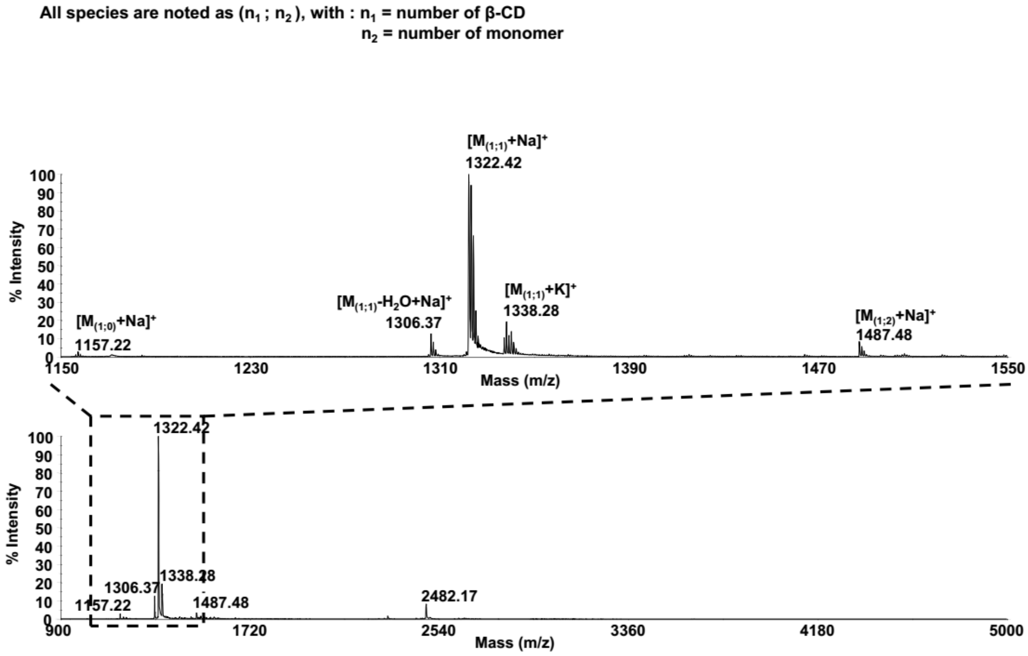

3. Neutron and X-ray scattering experiments

General Instrumentation

Small-angle X-ray scattering (SAXS). SAXS experiments were performed at the P12 BioSAXS beamline of the European Molecular Biology Laboratory (EMBL) at the storage ring PETRA III of the Deutsches Elektronen Synchrotron (DESY, Hamburg, Germany) using a Pilatus 2M detector (1475 × 1679 pixels; Dectris, Switzerland) and synchrotron radiation with a wavelength λ = 0.67 Å. The sample–detector distance was 4 m, allowing for measurements in the *q*-range interval from 0.005–0.65 $Å^{-1}$. The *q*-range was calibrated using the diffraction patterns of silver behenate. The experimental data were normalized to the incident beam intensity, corrected for non-

homogeneous detector response, and the background scattering of the DMF buffer was subtracted. Solutions have been placed in 1 mm glass capillaries. The temperature has been controlled by a Linkam heating stage HFSX 350 (Surrey, UK) 20 ± 0.1 °C. Twenty consecutive frames (each 0.05 s) comprising the measurement of the solvent (phosphate buffer pH 7.4) and sample were performed. In order to verify that no artefacts as a result of radiation damage occurred, all scattering curves of a recorded dataset were compared to a reference measurement (typically the first exposure) and finally integrated by automated acquisition program [6].

Small-angle neutron scattering (SANS). SANS experiments were performed on a KWS-1 SANS instrument of JCNS located at Heinz Maier-Leibnitz Zentrum (MLZ, Garching, Germany) [7]. The measurements were carried out at sample-to-detector distances of 8 and 1.5 m using a non-polarized incident beam with a neutron wavelength of λ = 5 Å and wavelength spread of $\Delta\lambda/\lambda$ = 10 %. The total covered *q*-range constituted 0.01–0.4 $Å^{-1}$. The samples were placed in 1 mm quartz cells and kept at 25 °C in a high-stability oven based on the Peltier effect. Corresponding DMF solution for background subtraction was measured along with the samples. A 1.5 mm thick flat piece of poly(methyl methacrylate) (PMMA) was used as a secondary absolute standard. Raw data were radially averaged and brought in absolute intensity units using QtiKWS10 software package [8].

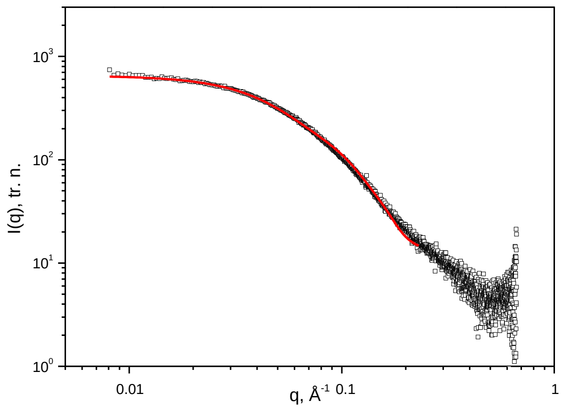

Figure S1: SAXS data of polymer **8** at 0.03 g/mL and fit by cylindrical model (red line).

*Cross section parameters of polymer **8***

On the length scales where the scattered intensity is controlled by the local stiffness of the polymer, it is possible to decouple the scattering into two contributions: one that originates from the whole length of chain and another that reflects the local cylindrical cross-section structure [9]. Scattering intensities at q > 0.03 Å^{-1} can be expressed as

$$I(\mathrm{q})/\mathrm{c} = \left(\frac{\pi}{q}\right)2\pi\int_0^\infty p_{CS}(r)J_0(qr)r\,dr = \left(\frac{\pi}{q}\right)I_{CS}(q)\ , \qquad (1)$$

where J_0 is the zeroth-order Bessel function and $I_{\mathrm{CS}}(q)$ is the cross-section scattering intensity. The normalized cross-section distance distribution function $p_{CS}(r)$ is given by [10].

$$p_{CS}(r) = \frac{1}{2\pi M_L}\int \Delta\rho(\mathbf{r}')\Delta\rho(\mathbf{r}+\mathbf{r}')\mathbf{r}'\,d\mathbf{r}'\ , \qquad (2)$$

where the vectors **r** and **r'** lay in the cross-section plane and M_L is the mass per unit length of polymer chain. We obtained an estimate of the distance distribution function $\tilde{p}_{CS}(r)$ by applying the IFT method. The distance distribution function exhibits a shape that is quite characteristic of an almost homogeneous cylindrical structure. We obtain a first estimate of the cross-section diameter from the maximum distance of approximately 30 Å (Supporting Information Figure S2).

From $\tilde{p}_{CS}(r)$ we can calculate the integral parameters of the micellar cross-section [11] such as the mass per length M_L and the cross-section radius of gyration $R_{g,CS}$, which is given by

$$R_{g,CS} = \left[\frac{\int_0^\infty r^2 \, p_{CS}(r) dr}{\int_0^\infty p_{CS}(r) dr} \right]^{1/2} . \tag{3}$$

The cross-section forward scattered intensity $I_{CS}(0)$ is given by

$$I_{CS}(0) = 2\pi \int_0^\infty p_{CS}(r) dr = \Delta\rho_m^2 M_L . \tag{4}$$

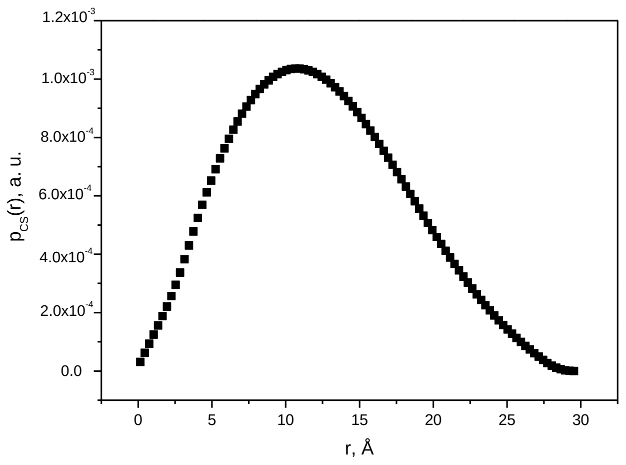

Figure S2: Pair distance distribution function of the cross-section obtained for 0.005 g/mL solution of polymer **8** (SANS data).

Check of flexibility

The range of the measured scattering vectors in SAS experiments corresponds to the length scale of the flexibility of the polymer chain. The SAS data gives a possibility for a direct observation of the flexibility using a Holtzer plot [12] with $qI(q)$ versus q (where q is the scattering vector and $I(q)$ is the scattering intensity) for representing the scattering data (Figure 3).

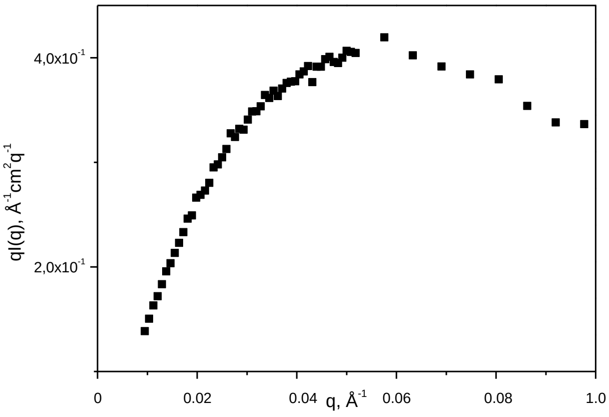

Figure S3: Holtzer representation of SANS scattering data for 0.01 g/ml solution of polymer **8** in DMF-d_7.

Modeling by model of stiff cylinders

The fitting of the SAS data has been performed by few different models depending on the expected shape of aggregates. It was assumed that there is no interaction between aggregates, which means the scattering intensities depend only on the size and shape of the aggregates. In this case the scattering intensities are written as [13]:

$$I(q) = n\langle |F(q)|^2 \rangle \qquad (5)$$

The values inside the brackets < > represent an average weighted by the distribution of particle sizes and orientations, n is the number density (corresponding to the

concentration) of the particles in the solution, *F(q)* is the amplitude of the form factor and is written as

$$F(q) = V\,\Delta\rho\, f(q,V,sh), \tag{6}$$

where *V* is the volume of the particle, $\Delta\rho$ is the contrast of scattering length densities between the particles and the solvent and the scattering function $f(q,V,sh)$ depends on the volume (size) and the shape, *sh*, of the particle.

In case of cylindrical particles *f(q,V,sh)* is expressed as

$$\mathrm{f(q,V,sh)} = \frac{\sin(qL/2\cos\beta)2J_1(qR\sin\beta)}{(qL/2\cos\beta)(qR\sin\beta)}\ , \tag{7}$$

where L is the length of the cylinder, R is the radius of the cylinder, J_1 is the first-order Bessel function, and β is the angle between the q-vector and the axis of the cylinder.

4. ITC Measurements

Isothermal titration calorimetry (ITC) was performed on a Nano ITC2G from TA Instruments at 25 °C.

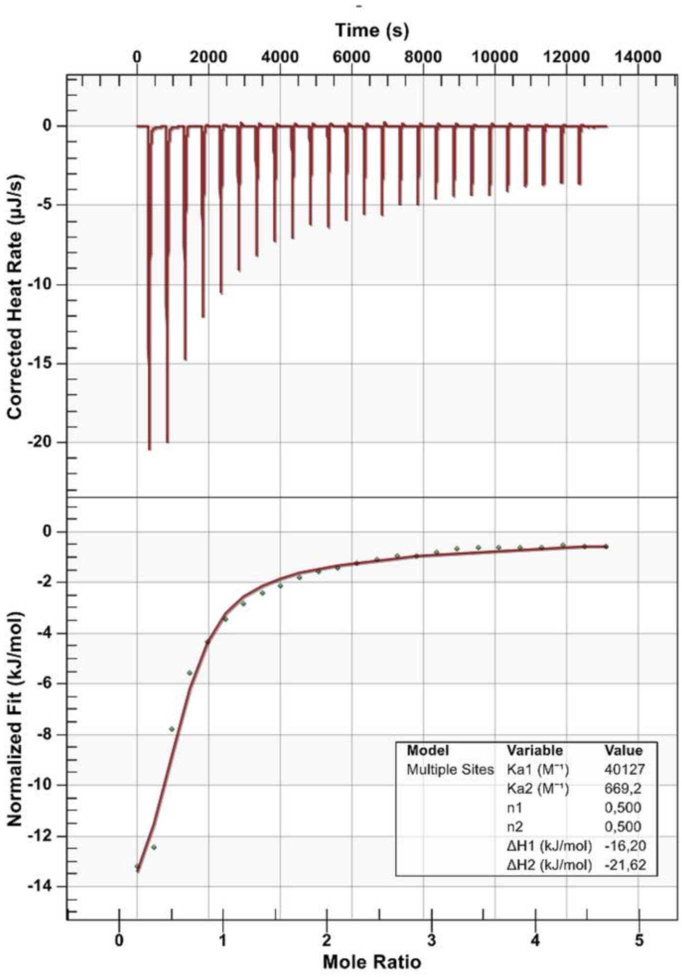

Figure S4: ITC curves of polymer **8** with adamantane hydrochloride **10**. Measurements were performed in phosphate buffer with 1% DMSO at pH 6.9 and 25 °C.

5. Surface preparation

Commercial available Si (111) wafers were used as substrates (Si-Mat, Kaufering, Germany). The surfaces were cleaned with piranha solution (3:2 mixture of sulfuric

acid and hydrogen peroxide) prior to surface functionalization to remove organic contamination. Subsequently the surface samples were rinsed with ultrapure water and dried with N_2. The silane monolayer was attached by vapor phase deposition of triethoxy(3-isocyanatopropyl)silane at a pressure of 2 mbar for 45 minutes. The silane triethoxy(3-isocyanatopropyl)silane was synthesized according to Yamamoto et al. [14]. After a washing procedure with THF and water the wafer remained either in 1 mM solution of mono(6-deoxy- 6-amino)-CD diluted in water or in 5 mM CD Polymer solution over night at room temperature. All surface samples were freshly prepared for the AFM measurements and stored in water for not more than three days. The silicon AFM tips (Nanosensors (TM) PPP-Cont AFM Probes, NanoandMore, Wetzlar, Germany) were functionalized in the same way as the surfaces. TEM image of the tip after experiments with CD polymers reveals the structural integrity of the tip (Figure 5). The material attached to the side of the tip is remanent from the dried solution.

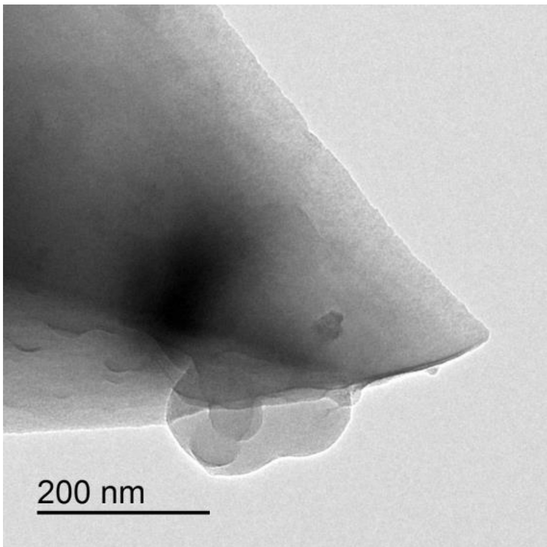

Figure S5: TEM image of the tip after experiments with CD polymers.

6. AFM measurements

The adhesive interaction between the different surface functionalizations was investigated by force spectroscopy measurements performed with a Nanowizard 3 setup (JPK Instruments, Berlin, Germany) in water or connector solution at room temperature. A connector molecule concentration of 10 µM was chosen in order to work in the saturation regime of the Langmuir curve (see [15] for more details). Silicon cantilevers with nominal normal spring constant of 0.2 N/m have been calibrated using the thermal noise analysis. To avoid surface damage and unspecific contributions to the adhesion force the maximum normal force was kept below 2 nN.
The force-distance curves were analyzed with the implemented Data Processing software (JPK instruments, Berlin, Germany). Each force curve was examined for the maximum pull-off force and the corresponding rupture length. The results were plotted in a histogram and the most probable pull-off force was obtained by a Gaussian curve fitted to the data. The shown adhesion forces represent average values of the pull-off forces obtained on at least three different surface positions, more than 100 force–distance curves each. The error bars reveal the standard deviation of the three surface positions. The rupture force of a single complex was obtained from the height of the last rupture event in force–distance curves. The height of the last rupture event was included in a histogram where the first peak reveals the most probable rupture force.

7. Other instrumentation parameters

All NMR spectra including ^{1}H and ^{13}C were measured at room temperature by a BrukerBioSpin spectrometer Magnet System 400 MHz Ultra shield plus (^{1}H:

400 MHz, ^{13}C: 100.6 MHz). The chemical shifts are given in parts per million (ppm) in relation to the corresponding solvent signal. The data analysis was performed with SpecManager included in ACDLabs 10.0 from Advanced Chemistry Development Inc., Toronto, Ontario, Canada. The multiplicities were assigned as follows: s for singlet, d for doublet, t for triplet, bs for broad signal and m for multiplet.

The solubility measurements were performed according to standard procedures [16]. Solutions of CD-polymer **8** (0-6 mM, 5 mL) in water were stirred with an excess of guest molecules **9** and **10** at 25 °C for 18 h. The resulting solutions were filtered through a Teflon (0.25 µm) syringe filter. The concentration of dissolved polymer **8** was determined by UV using its extinction coefficient ε = 14.800 M^{-1} cm^{-1}. The solubility of CD-polymer **8** was plotted versus the concentration of the corresponding guest molecules.

UV measurements were performed using an Evolution 220 UV–vis Spectrophotometer (Thermo Scientific), 1 mm quartz glass cuvettes (Hellma 110-QS) and ultrapure solvents. Fluorescence spectra were recorded in a Jasco Spectrofluorometer FP-6500 using ultrapure solvents. The concentrations of all solutions were in the micromolar range.

8. References

1. Bonifacio, M. C.; Robertson, C. R.; Jung, J.-Y.; King, B. T. *J. Org. Chem.* **2005**, *70*, 8522-8526.
2. Cocherel, N.; Rault-Berthelot, N.; Barrière, F.; Audebrand, N.; A. M. Z.; Slawin, Vignau, L. *Chem. Eur. J.* **2008**, *14*, 11328-11342.

3. Kretschmann, O.; Choi, S. W.; Miyauchi, M.; Tomatsu, I.; Harada, A.; Ritter, H. *Angew. Chem.* **2006**, *118*, 4468-4472; *Angew. Chem. Int. Ed.* **2006**, *45*, 4361-4365.

4. Wenz, G.; Strassnig, C.; Thiele, C.; Engelke, A.; Morgenstern, B.; Hegetschweiler, K. *Chem. Eur. J.* **2008** *14*, 7202-7211.

5. Przybylski, C.; Blin, F.; Jarroux, N. *Macromolecules* **2011**, *44*, 1821-1830.

6. Franke, D.; Kikhney, A. G.; Svergun, D. I. Automated acquisition and analysis of small angle X-ray scattering data. *Nuclear Instruments and Methods in Physics Research Section A: Accelerators, Spectrometers, Detectors and Associated Equipment* **2012**, *689*, 52-59.

7. Feoktystov, A. F.; Frielinghaus, H.; Di, Z.; Jaksch, S.; Pipich, V.; Appavou, M. S.; Babcock, E.; Hanslik, R.; Engels, R.; Kemmerling, G.; Kleines, H.; Ioffe, A.; Richter D.; Brückel T. *J. Appl. Cryst.* **2015**, *48*, 61-70.

8. www.qtikws.de.

9. Schurtenberger, P., Jerke, G., Cavaco, C., Pedersen, J.S. *Langmuir* **1996**, *12*, 2433-2440.

10. Feigin, L. A.; Svergun, D.I. *Structure Analysis by Small-Angle X-Ray and Neutron Scattering*; Plenum Press: New York, 1987: pp 47-48.

11. Glatter, O. In *International Tables for Crystalography*; Wilson, A. J.C., Eds; Kluwer Academic Publishers: Dordrecht, 1992; Vol. C, pp 89-105.

12. Denkinger, P.; Burchard, W. *J. Polym. Sci.B: Polym. Phys.* **1991**, *29*, 589-600.

13. Pedersen, J. S. *Adv. Coll. Inter. Sci.* 1997, *70*, 171-210.

14. Yamamoto, T.; Terada, A.; Muramatsu, T.; Ikeda, K. *Org. Prep. Proced. Int.* **1994**, *26*, 555-557.

15. Blass, J.; Albrecht, M.; Bozna, B. L.; Wenz, G.; Bennewitz, R. *Nanoscale* **2015**, *7*, 7674-7681.

16. Kang, J.; Kumar, V.; Yang, D.; Chowdhury, P. R.; Hohl, R. *J. Eur. J. Pharm. Sci.* **2002**, *15*, 163-170.
